# Alcohol consumption among patients diagnosed with genitourinary cancers

**DOI:** 10.1002/bco2.70086

**Published:** 2025-09-18

**Authors:** Aidan Weitzner, Carlos Rivera Lopez, Joseph Cheaib, Michelle Higgins, Nirmish Singla

**Affiliations:** ^1^ Department of Urology, Brady Urological Institute Johns Hopkins University School of Medicine Baltimore MD USA; ^2^ Department of Oncology Johns Hopkins University School of Medicine Baltimore MD USA

**Keywords:** alcohol, genitourinary cancer, modifiable behaviour

## Abstract

**Objective:**

To characterize alcohol consumption and binge‐drinking patterns among individuals with GU cancers (prostate, kidney, bladder and testicular) compared to a propensity‐matched cohort without cancer in a large, nationally diverse population.

**Materials and Methods:**

We conducted a retrospective, cross‐sectional study utilizing data from the National Institutes of Health *All of Us* Research Program. Matching accounted for age, sex assigned at birth, smoking status, comorbidities and education/marital status. The primary outcome was self‐reported drinking frequency. The secondary outcomes were self‐reported binge‐drinking frequency and Alcohol Use Disorders Identification Test (AUDIT‐C) scores.

**Results:**

Drinking and binge‐drinking among individuals with GU malignancy (N = 11 522) closely resembled those of matched controls (N = 47 747), with the majority (53%) consuming at least 2–4 drinks per month. There was no significant association between GU cancer diagnosis and increased drinking frequency (OR: 0.99; p = 0.65), binge‐drinking frequency (OR: 0.85; p: 0.055) or AUDIT‐C (OR: 0.99; p =0.65). Individuals diagnosed with kidney cancer had reduced odds of higher alcohol use (OR: 0.76; p < 0.001) and AUDIT‐C score (OR: 0.83; p < 0.001) compared to controls.

**Conclusion:**

In this large cohort, including traditionally underrepresented minorities, alcohol use was highly prevalent among those with GU malignancies. Drinking behaviours were similar to individuals without cancer, underscoring the need for integration of lifestyle‐focused interventions into survivorship care, as alcohol remains a common and modifiable behaviour with wide‐ranging health implications.

## 1 INTRODUCTION

A recent statement by the U.S. Surgeon General has refocused public and scientific discourse on the growing public health burden of alcohol use, particularly its role in cancer development.^1^ Epidemiological evidence has established that alcohol consumption is causally linked to malignancies of the head and neck (oral, pharyngeal, laryngeal), breast, liver and colorectum.^2^, ^3^, ^4^ In contrast to the dose‐dependent association observed in several cancer types, the relationship between alcohol use and genitourinary (GU) malignancies is less clear. Existing evidence has yielded inconsistent findings; some studies report weak or null associations,^5^, ^6^ while others suggest protective or deleterious effects depending on the cancer subtype.^7^, ^8^

The current body of literature examining alcohol use and GU malignancies is limited by both methodological constraints and narrow population selection. Many conclusions are drawn from meta‐analyses, which, while valuable for generating epidemiologic insights, often conflate results by aggregating studies with heterogenous designs, inconsistent definitions of alcohol exposure and varied levels of confounder control. Additionally, most studies have focused on the relationship between alcohol consumption and incident cancer risk, with far fewer exploring post‐diagnosis alcohol use.^9^ No large‐scale investigations have specifically evaluated post‐diagnosis behaviours for those diagnosed with GU malignancy. This gap is particularly concerning, given rising survivorship. There remains a critical need for contemporary data that capture alcohol use behaviours across the GU cancer care continuum, particularly among individuals from traditionally underrepresented backgrounds.

In this context, we sought to characterize alcohol use patterns among individuals diagnosed with GU malignancies compared to a matched cohort without cancer. By leveraging a large, diverse population dataset from *All of Us*, this study aims to provide clearer insight into alcohol behaviours across the GU cancer care continuum and address persistent gaps in the literature. Given that alcohol consumption patterns tend to remain stable over time, our analysis may also offer preliminary insight into pre‐diagnosis behaviour.^10^

## 2 MATERIALS AND METHODS

### 2.1 Study Design

We conducted a cross‐sectional analysis of data from the *All of Us* Research Program, a National Institutes of Health initiative integrating participant data from surveys and electronic health records (EHR), with an emphasis on populations traditionally underrepresented in biomedical research.^11^ Researchers acquired participant‐level data through the *All of Us* data passport and adhered to the *All of Us* Data Use and Registration agreement. This study followed the Strengthening the Reporting of Observational Studies in Epidemiology (STROBE) reporting guidelines.^12^

### 2.2 Study Cohort

The Controlled Tier Dataset v8 was utilized to identify all individuals diagnosed with the major GU malignancies (pan‐GU), abstracted from EHR using the International Classification of Diseases, 10th edition codes for malignant neoplasm of bladder (C67), prostate (C61), kidney (C64) and testis (C62).^13^ Controls were individuals participating in *All of Us* without a documented GU malignancy diagnosis. Patients were excluded if they had: unknown GU malignancy status, incomplete alcohol use data from the *All of Us* 'Lifestyle' survey, missing covariates for propensity‐based matching, or assigned female‐at‐birth designation with EHR diagnosis of prostate and testicular malignancy. Demographic data (i.e. marital, education status) were extracted from the *All of Us* 'Basics' survey (Supplement 1).

### 2.3 Propensity Scores and Matching

Propensity scores were estimated using logistic regression, incorporating age at survey completion, self‐reported sex at birth (if applicable), current smoking status and comorbidities (type 2 diabetes mellitus, hypertension, obesity).^14^ We performed a 1:4 case‐control, nearest‐neighbour matching without replacement for the pan‐GU cohort and each cancer subtype. A calliper width of 0.2 times the standard deviation of the logit‐transformed propensity score was used for matching criteria. Matching was conducted without replacement, and a standardized mean difference (SMD) of 0.1 or greater was considered a meaningful imbalance.

### 2.4 Outcome Variables

The primary outcome was alcohol consumption frequency over the past year, assessed in the *All of Us* ‘Lifestyle’ survey. Participants categorized themselves in the following ordinal categories: never, monthly or less, 2–4 times per month, 2–3 times per week, 4 or more times per week. The secondary outcome was binge‐drinking frequency (6 + drinks) within the past year, self‐reported as: never, less than monthly, monthly, weekly, daily. The Alcohol Use Disorders Identification Test (AUDIT‐C) was calculated as a composite measure, accounting for quantity, frequency and binge‐drinking.^15^ Outcomes were compared between propensity‐matched cohorts, with a sub‐analysis for each GU malignancy subtype.

### 2.5 Subgroup Definitions

To better characterize pre‐diagnosis behaviours, we conducted separate subanalyses among individuals diagnosed within 2 and within 12 months of survey completion, given that the survey assessed drinking behaviours over the preceding 12 months. Each subcohort was compared to matched controls who completed the *All of Us* survey during the same time interval.

### 2.6 Statistical Analysis

Descriptive statistics by malignancy status were presented as proportions for categorical variables and medians (IQR) for continuous variables. Outcomes of alcohol use were treated as ordinal dependent variables. Ordinal logistic regression was used to assess the association between outcomes (alcohol consumption frequency, binge‐drinking frequency, AUDIT‐C) and GU malignancy diagnosis, adjusting for age, race, gender (if applicable), smoking status, comorbidities (diabetes mellitus type 2 [DMT2], hypertension, obesity) and marital and education status. This model was used to calculate the odds ratio (OR) and 95% confidence interval (CI), with non‐malignancy as the reference group. Spearman's rank correlation was performed to assess a monotonic relationship between time since diagnosis and alcohol consumption frequency. Statistical significance was set at <0.05. All statistical analyses were performed in R Studio (version 4.2.3) via the *All of Us* Researcher Workbench Cloud Platform.

## 3 RESULTS

**FIGURE 1:**
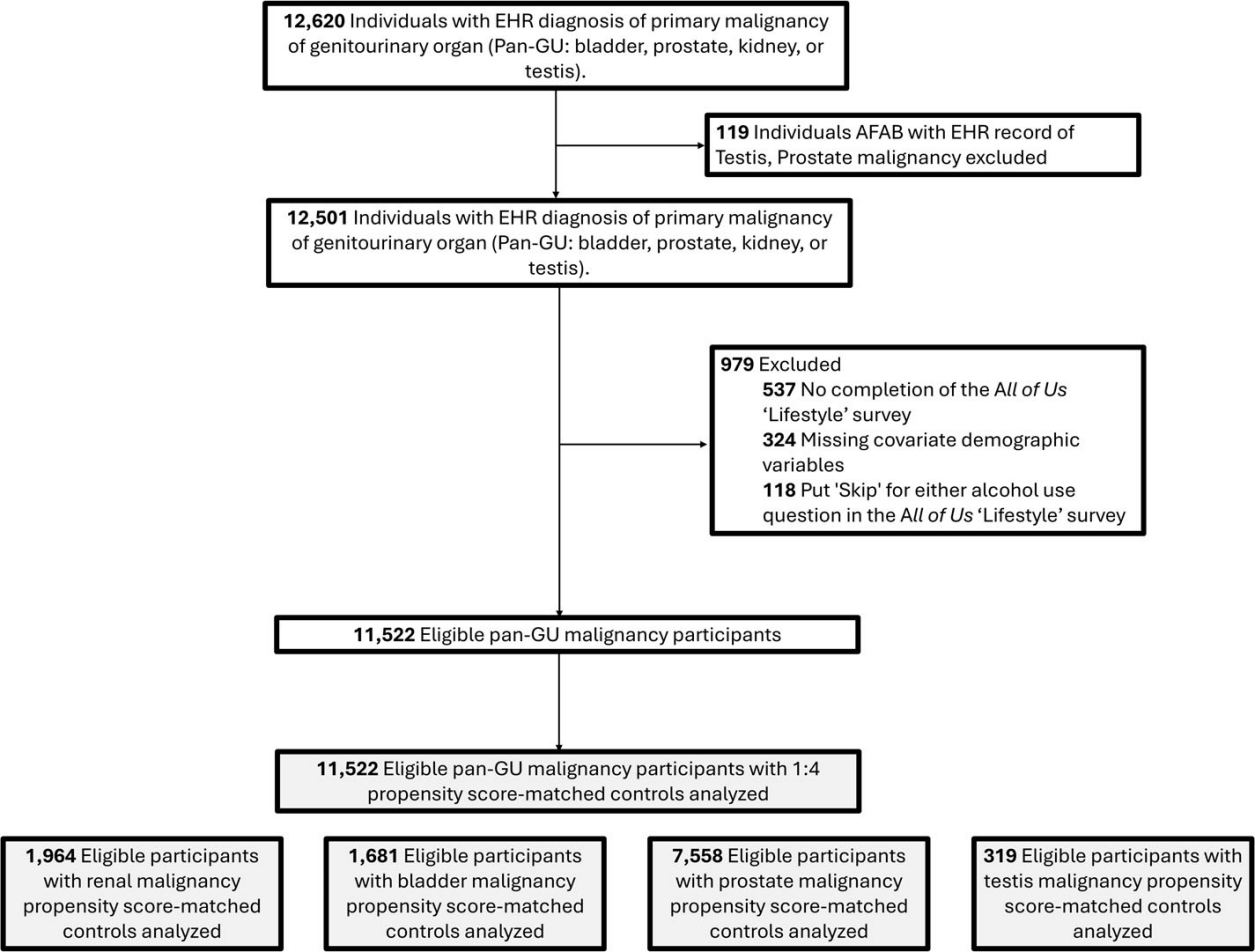
Study inclusion flowchart of *All of Us* participants diagnosed with a GU malignancy.

A total of 12,620 individuals diagnosed with a GU malignancy were identified (Figure 1). Of these, 1,098 (8.7%) were excluded due to sex at birth not aligning with diagnosis (n = 119), no survey completion (n = 537), missing covariates (n = 324) or selection of 'Skip' for alcohol use questions (n = 118). The final cohort included 11,522 individuals matched to 44,747 controls. Prostate (n = 7558), kidney (n = 1964), bladder (n = 1681) and testis (n = 319) cancer comprised the primary malignancy types. Baseline characteristics are summarized in Table 1. The GU malignancy cohort was older (median (IQR): 70 (63,76)) than the control cohort (median (IQR): 69 (63,75), p < 0.001). Groups were well matched on gender (p:0.53), race (p:0.31) and ethnicity (p:0.69). Smoking status and comorbidities (obesity, hypertension, depression, diabetes) were also comparable between the two groups.

**TABLE 1:** Demographic and clinical characteristics of *All of Us* participants diagnosed with a GU malignancy compared to a 1:4 propensity‐matched noncancer cohort..

Demographic Variables	Patients, No (%)		P^1^	SMD
	GU Cancer (N = 11 522)	No GU Cancer (N = 47 747)		
**Age (Median, IQR)**	70 (63, 76)	69 (63, 75)	<0.001	0.056
**Time since Diagnosis (Months)**	34 (2, 90)			
**Assigned Male at Birth**	9992 (87)	38 653 (86)	0.53	0.021
**Hispanic Ethnicity**	935 (8)	3660 (8)	0.69	0.015
**Race**			0.31	0.035
White	8433 (73)	33 240 (69)		
African American	1380^12^	5048 (11)		
Other (Asian, American Indian, Middle Eastern, Native Hawaiian)	1088^10^	4201 (9)		
None of These	621 (5)	5258 (11)		
**Highest Education Level**			<0.001	0.093
Advanced Degree	3744 (33)	13 143 (29)		
College	5565 (49)	21 909 (46)		
High School/GED	1967^16^	8595 (16)		
Less than High School	246 (2)	4100 (9)		
**Marital Status**			<0.001	0.075
Married/Living with Partner	7661 (67)	28 387 (63)		
Divorced/Separated	1776^15^	7429 (17)		
Widowed	852 (7)	3538 (8)		
Never Married	1061^9^	4785 (11)		
Other	172 (2)	608 (1)		
**Current Smoker**	1023^17^	3925 (17)	0.24	0.039
**Obesity**	3190 (28)	12 326 (28)	0.77	0.003
**Morbid Obesity**	1171^10^	4849 (11)	0.039	0.022
**Diabetes Mellitus Type 2**	3523 (31)	14 052 (31)	0.09	0.018
**Depression**	1104^10^	4263 (10)	0.87	0.002
**Hypertension**	8185 (71)	31 660 (71)	0.56	0.006

1. Comparative statistics of demographic variables for the propensity‐matched cohort. Wilcoxon Rank‐Sum test for continuous variables. Chi‐squared test for all categorical variables.

The distribution of drinking frequency, binge‐drinking (6 + drinks) frequency and AUDIT‐C score by GU malignancy is presented in Table 2. The composition is shown for the pan‐GU cohort, as well as each GU malignancy subtype. Among individuals with a GU malignancy, time since diagnosis was not significantly associated with drinking frequency (Spearman's rho: −0.004, p: 0.64). Similar findings were observed across malignancy subtypes, with no significant correlation between time since diagnosis and drinking frequency in prostate (rho: 0.0024, p: 0.85), kidney (rho: −0.028, p: 0.29), bladder (rho: 0.034, p: 0.23) and testis (rho: −0.02, p: 0.73) cancer.

**TABLE 2:** Alcohol consumption patterns among *All of Us* participants among pan‐GU and GU cancer subtypes compared to 1:4 propensity‐matched noncancer cohort.

	Pan‐GU Cancer Cohort	Noncancer Cohort	OR (95% CI)^1^	Prostate Cancer	No Prostate Cancer	OR (95% CI)^1^	Kidney Cancer	No Kidney Cancer	OR (95% CI)^1^	Bladder Cancer	Noncancer Cohort	OR (95% CI)^1^	Testis Cancer	No Testis Cancer	OR (95% CI)^1^
**Drinking Frequency**			0.99 (0.93, 1.05)			1.03 (0.96, 1.11)			0.76 (0.65, 0.90)*			1.04 (0.89, 1.24)			0.98 (0.64, 1.50)
*Never*	2570 (22)	10 388 (24)		1553^21^	6783 (22)		528 (27)	1976^26^		433 (26)	1535^23^		56 (18)	212 (17)	
*Monthly or Less*	2961 (26)	11 345 (26)		1749^23^	6953 (23)		665 (34)	2496 (32)		468 (28)	1895^28^		79 (25)	376 (30)	
*2–4 per Month*	2032 (18)	7996 (18)		1350^18^	5360 (18)		331 (17)	1315^17^		279 (17)	1142^17^		72 (23)	242 (19)	
*2–3 per Week*	1678 (15)	6415 (14)		1207^16^	4483 (15)		228 (12)	999 (13)		183 (11)	902 (14)		60 (19)	215 (17)	
*4 or Greater per Week*	2220 (20)	8355 (19)		1657^22^	6164 (21)		203 (10)	1040^13^		309 (19)	1216^18^		51 (16)	225 (18)	
**Binge‐Drinking Frequency**			0.85 (0.71, 1.01)			0.87 (0.71, 1.07)			0.90 (0.59, 1.38)			0.76 (0.47, 1.23)			0.64 (0.24, 1.71)
*Never*	6286 (71)	23 498 (70)		4231 (72)	16 147 (71)		1004 (71)	3914 (68)		917 (75)	3737 (73)		134 (51)	504 (48)	
*< Monthly*	1721 (20)	6673 (20)		1132^19^	4304 (19)		286 (20)	1225^21^		223 (18)	918 (18)		80 (31)	319 (30)	
*Monthly*	468 (5)	2002^6^		317 (6)	1288^6^		76 (5)	368 (6)		50 (4)	252 (5)		25 (10)	119 (11)	
*Weekly*	242 (3)	1078^3^		168 (3)	685 (^3^		<35	179 (3)		<25	120 (2)		<20	78 (7)	
*Daily*	110 (1)	551 (2)		77 (1)	368 (2)		<20	76 (1)		<20	77 (2)		<20	28 (3)	
**AUDIT‐C**	2 (1,4)	2 (1,4)	0.99 (0.94, 1.03)	2 (1,4)	2 (1,4)	0.98 (0.91, 1.06)	2 (0,3)	1 (0,3)	0.83 (0.75,0.92)*	2 (0,3)	2 (1,4)	0.93 (0.83, 1.04)	2 (1,4)	2.5 (1,4)	0.96 (0.73, 1.27)

*1. Odds ratio (OR) and 95% confidence interval (CI) were calculated from ordinal logistic regression, with alcohol use variables treated as an ordinal dependent variable. Regression was adjusted for age, race, gender, smoking status, DMT2, hypertension, obesity, education status and marital status*.

*Reported kidney cancer was associated with lower odds of higher consumption of alcohol and higher AUDIT‐C score.

**FIGURE 2:**
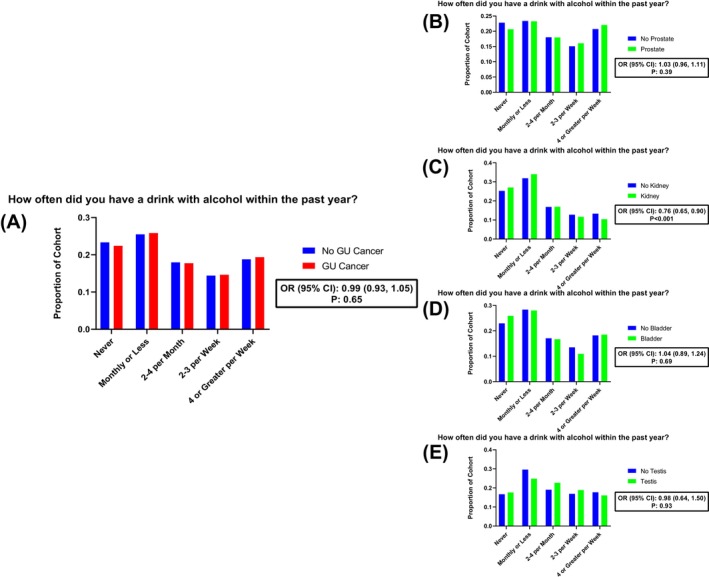
Distribution of drinking frequency across genitourinary malignancy subtypes compared to propensity‐matched noncancer controls. Bar plots show the proportion of individuals reporting different levels of alcohol consumption within the last year, stratified by A) pan‐GU cancers, B) prostate, C) kidney, D) bladder and E) testis cancer. Ordinal logistic regression models adjusted by demographic and clinical characteristics were used to report odds ratio (OR) and 95% confidence intervals (CIs) of more frequent alcohol consumption. C) A diagnosis of kidney cancer was associated with significantly lower odds of reporting heavier drinking (OR (95% CI): 0.76 (0.65,0.90); p < 0.001).

**FIGURE 3:**
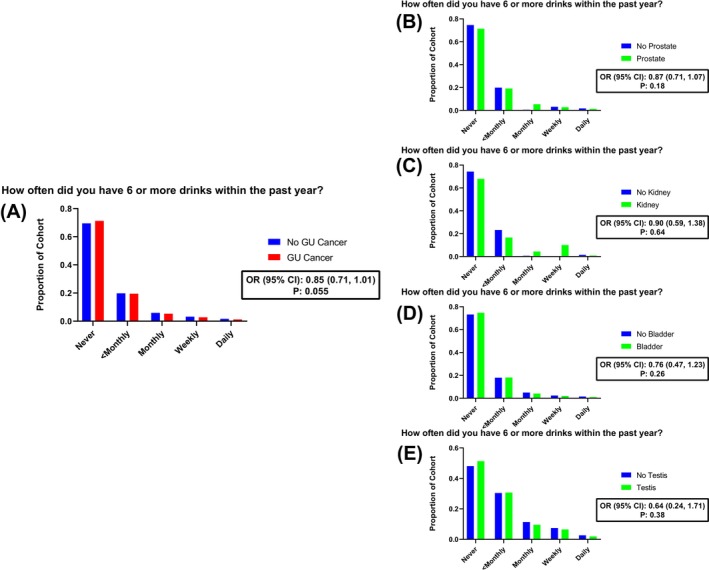
Distribution of binge‐drinking frequency across genitourinary malignancy subtypes compared to propensity‐matched noncancer controls. Bar plots show the proportion of individuals reporting different levels of binge‐drinking within the last year, stratified by A) pan‐GU cancers, B) prostate, C) kidney, D) bladder and E) testis cancer. Ordinal logistic regression models adjusted by demographic and clinical characteristics were used to report odds ratio (OR) and 95% confidence intervals (CIs) more frequent heavy alcohol consumption.

For each cohort, the proportion of participants in each drinking and binge‐drinking frequency category is visualized in Figures 2 and 3, respectively. Across all GU malignancies, the distribution of drinking frequency closely mirrored that of the matched control cohort, with the majority (53%) stating that they consume 2–4 drinks per month or more. Respondents diagnosed with a GU malignancy did not demonstrate increased drinking frequency (OR (95% CI): 0.99 (0.93, 1.05); p: 0.65) or binge‐drinking frequency (OR (95% CI): 0.85 (0.71, 1.01); p: 0.055) compared to individuals with no malignancy. Similarly, composite AUDIT‐C scores did not differ significantly between pan‐GU cancer and matched‐control cohorts (OR (95% CI): 0.99 (0.94, 1.03); p:0.65).

Among individual malignancies, no significant associations were observed between prostate cancer patients and reported drinking frequency (OR (95% CI): 1.03 (0.96, 1.11); p: 0.39), binge‐drinking frequency (OR (95% CI); 0.87 (0.71, 1.07); p: 0.18), or AUDIT‐C score (OR (95% CI): 0.98 (0.91, 1.06); p:0.87). Similar patterns were observed for bladder cancer (drinking frequency OR (95% CI): 1.04 (0.89, 1.24), p: 0.69; binge‐drinking OR (95% CI): 0.76 (0.47, 1.23), p: 0.26; AUDIT‐C OR (95% CI): 0.93 (0.83, 1.04), p: 0.77) and testis cancer (drinking frequency OR (95% CI): 0.98 (0.64, 1.50), p: 0.93; binge‐drinking OR (95% CI): 0.64 (0.24, 1.71), p: 0.38; AUDIT‐C OR (95% CI): 0.96 (0.73, 1.27), p: 0.89). Although not significantly different from the matched‐control cohort, the proportion of participants reporting any binge‐drinking was highest among individuals with testicular cancer (49%) relative to other GU malignancies.

Individuals with kidney cancer demonstrated lower odds of increased drinking frequency (OR (95% CI): 0.76 (0.65, 0.90); p < 0.001) compared to participants with no cancer. This finding was supported by the AUDIT‐C analysis, in which individuals with kidney cancer had significantly reduced odds of higher AUDIT‐C scores (OR (95% CI): 0.83 (0.75, 0.92), p < 0.001). However, there was no significant difference observed in binge‐drinking frequency (OR (95% CI): 0.90 (0.59, 1.38); p: 0.64).

In the subanalysis of 432 individuals with GU malignancy who completed the preceding 12‐month alcohol use survey within 2 months of diagnosis, thereby likely capturing their pre‐diagnosis alcohol use patterns, there was no difference in drinking frequency (OR (95% CI): 0.93 (0.66, 1.31); p:0.34), binge‐drinking frequency (OR (95% CI): 1.40 (0.96, 2.04); p:0.34), or AUDIT‐C score (OR (95% CI): 0.95 (0.68, 1.33); p:0.34) compared to 1726 matched controls (Supplementary Table 2). A similar lack of association across all alcohol use outcomes was observed in the cohort of patients diagnosed within 12 months of survey completion (Supplementary Table 1).

## 4 DISCUSSION

Leveraging data from a large, nationally representative cohort, our study provides novel insight into alcohol consumption patterns among patients diagnosed with GU malignancies. We found that alcohol use was highly prevalent among individuals with GU cancer and that post‐diagnosis drinking frequency, binge‐drinking frequency and AUDIT‐C did not differ meaningfully from matched peers without cancer. To our knowledge, this is the largest contemporary study to evaluate post‐diagnosis alcohol consumption patterns across prostate, kidney, bladder and testicular cancer populations.

Similar to the findings by Shi et al across cancer survivors more broadly,^9^ we found that alcohol consumption remains common following a GU cancer diagnosis, mirroring that of matched peers. Similarly, we also observed that the time since diagnosis was not associated with a reduction in alcohol consumption across any GU malignancy subtype, suggesting that the cancer diagnosis alone may not be sufficient to alter drinking behaviour. Although prior studies indicate a general trend of stability in alcohol consumption, this conclusion is limited by not knowing baseline consumption patterns prior to diagnosis.^10^, ^16^ Nonetheless, given prior evidence linking post‐diagnosis alcohol use to worse outcomes in other cancer populations, our findings may raise important concerns about persistent risky behaviours in GU cancer survivorship.^17^, ^18^ This underscores a critical need for structured lifestyle counselling and alcohol‐related screening in post‐treatment care pathways.

The lack of association between alcohol consumption frequency and GU cancer diagnosis aligns with prior evidence suggesting a weak or null relationship. While alcohol use across a pan‐GU population has not been previously evaluated, several investigations into individual GU malignancies have reported similar findings. For instance, prior meta‐analyses and cohort studies have shown there was no significant association between alcohol intake and bladder cancer.^19^, ^20^ Our findings suggest that this null relationship persists after diagnosis. Similarly, multiple large‐scale studies have demonstrated no relationship between prostate cancer and increased alcohol consumption, including analyses focused on advanced or lethal disease.^21^, ^22^ Notably, as testicular cancer accounts for less than 1% of male tumours, lifestyle studies behaviour data remain limited, although our results contribute to new evidence in this underexplored area.^23^

Interestingly, kidney cancer was the only GU malignancy in which post‐diagnosis alcohol consumption frequency was lower compared to controls. Prior studies have reported modest protective associations between alcohol consumption and kidney cancer risk.^24^, ^25^ However, the biological rationale remains speculative.^26^ While our findings may be consistent with the notion that alcohol use is a protective factor, it is important to note that our data mostly reflect alcohol consumption after diagnosis and may not necessarily represent pre‐diagnosis alcohol consumption patterns. Furthermore, the clinical relevance of this modest association is unlikely to outweigh the overall risks of alcohol consumption, especially in a population of cancer survivors in whom long‐term health optimization is critical. More prospective studies are needed to clarify the timing, mechanisms and health implications of alcohol use in patients with kidney cancer.

When examining binge‐drinking behaviours, defined as consuming six or more drinks on one occasion, we found no significant association between increased post‐diagnosis binge‐drinking frequency and GU cancer overall. Similar null associations were observed across all malignancy subtypes. Although a smaller proportion (30%) of the cohort binged alcohol compared to overall alcohol use (76%), the comparable rates of binge‐drinking between cancer survivors and matched controls suggest high‐risk drinking persists after diagnosis. This was similar to previous studies across cancer more generally.^9^, ^10^ Individuals with testicular cancer had the highest rates of binge drinking (49%) among GU cancer subtypes, although comparable to matched controls. This likely reflects the younger demographic of this group, as this population exhibits the highest rate of binge‐drinking.^27^ This finding highlights the importance of tailoring survivorship needs for younger adult populations, with a focus on reduction of harmful alcohol use.

Although our data mostly reflect post‐diagnosis behaviour, we analysed a subset of individuals who completed the alcohol use survey shortly after receiving a GU cancer diagnosis to potentially gain insight into pre‐diagnosis patterns, given the survey captures data on alcohol consumption over the preceding 12 months. In this subcohort analysis of patients who answered the survey within 2 months of diagnosis, there were no significant differences in drinking frequency, binge‐drinking frequency or AUDIT‐C scores between cancer cases and matched controls. These findings suggest that alcohol consumption patterns were broadly similar in the immediate pre‐diagnosis period. The consistency of these findings in the broader subcohort analysis of patients who completed the survey within 12 months of diagnosis further supports the interpretation that alcohol use behaviours do not substantially differ prior to GU cancer diagnosis. When taken in the context of prior literature demonstrating the chronicity and stability of alcohol use patterns over time,^28^ these subanalyses, although unable to establish temporality, suggest that alcohol consumption is unlikely to be a major behavioural precursor to GU malignancy. Nevertheless, prospective studies with repeated alcohol use assessments are needed to definitively determine causality.

This study has several inherent limitations. The *All of Us* survey instrument to assess alcohol use was limited to self‐reported, post‐diagnosis AUDIT‐C and may introduce recall, social desirability or reporting bias. We were unable to draw granular conclusions, such as differentiating consumption of alcohol types (beer, wine, liquor) that may have differing health impacts. Further research should focus on the collection and integration of standardized alcohol screening tools, such as the CAGE questionnaire, supplemented with detailed assessments of drinking patterns and beverage types to enhance measurement accuracy and comparability across studies.^15^, ^29^ Due to the cross‐sectional study design and the nature of data available in the survey, assessment of pre‐diagnosis alcohol use to evaluate for temporality, causality and behaviour modification with regards to the diagnosis of a GU malignancy could not be performed, and changes in alcohol use before versus after cancer diagnosis could not be evaluated. The relationship between alcohol consumption and aggressiveness of cancer or tumour growth rates was unable to be evaluated as well. Additionally, because cancer diagnoses were abstracted from EHR, there is potential for misclassification or data entry errors, as illustrated in the exclusion of female‐at‐birth participants with a diagnosis of prostate or testis cancer. Although propensity‐score matching was used to adjust for key covariates, residual confounding is possible. Future longitudinal research with validated exposure metrics and granular clinical detail is needed to better characterize alcohol consumption across the GU cancer care continuum.

## 5 CONCLUSION

In this cross‐sectional, nationally representative study of individuals with genitourinary malignancies, we found that alcohol consumption remains highly prevalent after diagnosis, with drinking and binge‐drinking frequency closely resembling that of matched peers without cancer. Time since diagnosis was not associated with drinking behaviour, suggesting diagnosis alone may not prompt a reduction in alcohol use. Across prostate, bladder and testicular cancer there was no association between drinking outcomes and diagnosis, while individuals with kidney cancer reported modestly lower alcohol use compared to matched controls. Although alcohol may have a limited association with GU cancer risk, its continued prevalence post‐diagnosis underscores the importance of integrating lifestyle‐focused care pathways, as alcohol remains a modifiable behaviour with well‐established health risks.

### 5.1 Take Home Message

Alcohol consumption patterns after genitourinary cancer diagnosis are similar to matched controls. As alcohol is associated with numerous health risks, there is a need to integrate lifestyle discussions into the cancer care continuum.

## AUTHOR CONTRIBUTIONS

**Aidan Weitzner:** Conceptualization; methodology; formal analysis; writing—original draft; writing—review and editing. **Carlos Rivera Lopez:** Conceptualization; methodology; formal analysis; writing—review and editing. **Joseph Cheaib:** Methodology; formal analysis; writing—review and editing. **Michelle Higgins:** Writing—review and editing. **Nirmish Singla:** Conceptualization; methodology; formal analysis; writing—original draft; writing—review and editing; supervision; project administration.

## CONFLICT OF INTEREST STATEMENT

The authors have no conflicts of interest to disclose.

## Supporting information


**Data S1.** Supporting Information

**Supplementary Table 1.** Alcohol consumption patterns in the 12‐month post‐diagnosis sub‐cohort among pan‐GU and GU cancer subtypes compared to noncancer controls.

**Supplementary Table 2.** Alcohol consumption patterns in the two‐month post‐diagnosis subcohort: Pan‐GU cancer versus noncancer controls.
