# Supplementary material for: Alcohol consumption among patients diagnosed with genitourinary cancers
Source: BJUI Compass. 2025 Sep 18;6(9):e70086. doi: 10.1002/bco2.70086 (PMC12446081; doi:10.1002/bco2.70086)
Supplement: Supplementary file 1 — Data S1. Supporting Information [file BCO2-6-e70086-s001.pdf]

# The Basics

This survey asks questions about you, your work, and your home life. This is to better understand how they may affect health. To ensure your privacy, your name will be separated from your answers before they are shared with researchers.

It takes about 10-15 minutes to answer these questions. Please answer each question as honestly as possible. There are no right or wrong answers to any of the questions. It is important that you answer as many questions as you can. We are looking for your own answers, and not what you think your doctors, family, or friends want you to say.

Don't feel like you have to spend a long time on each question. The first answer that comes to you is usually the best one. If you aren't sure how to answer a question, choose the best answer from the options given.

---

**The first 9 questions ask about basic background information.**

**In what country were you born?**[1]
[Original source question text: In what country {were you/was NON-SP Head} born?]
- USA
- Other (free text)

**Which categories describe you? Select all that apply. Note, you may select more than one group.**[2]
[Original source question text: Which categories describe Person 1? Mark all boxes that apply AND print details in the spaces below. Note, you may report more than one group.]
- ❑ American Indian or Alaska Native (For example: Aztec, Blackfeet Tribe, Mayan, Navajo Nation, Native Village of Barrow (Utqiagvik) Inupiat Traditional Government, Nome Eskimo Community, etc.)
  [Original source question text: American Indian or Alaska Native – Print name of enrolled or principal tribe(s), for example, Navajo Nation, Blackfeet Tribe, Mayan, Aztec, Native Village of Barrow Inupiat Traditional Government, Nome Eskimo Community, etc.]
  *Branching Logic: when "American Indian or Alaska Native" selected, then:*
  - ❑ American Indian
    *Branching Logic: when "American Indian" selected, then:*
      Provide the name of the tribe in which you are enrolled or affiliated or your tribal descent (For example: Aztec, Blackfeet Tribe, Mayan, Navajo Nation, Native Village of Barrow (Utqiagvik) Inupiat Traditional Government, Nome Eskimo Community, etc.)
      **Please specify.**[2]
      - *(display optional free text)*
  - ❑ Alaska Native

*Branching Logic: if "Alaska Native" selected, then:*

Provide the name of the tribe in which you are enrolled or affiliated or your tribal descent (For example: Aztec, Blackfeet Tribe, Mayan, Navajo Nation, Native Village of Barrow (Utqiagvik) Inupiat Traditional Government, Nome Eskimo Community, etc.)

**Please specify.**[2]

- (*display optional free text*)

- ❏ Central or South American Indian

  *Branching Logic: when "Central or South American Indian" selected, then:*

  Provide the name of the tribe in which you are enrolled or affiliated or your tribal descent (For example: Aztec, Blackfeet Tribe, Mayan, Navajo Nation, Native Village of Barrow (Utqiagvik) Inupiat Traditional Government, Nome Eskimo Community, etc.)

  **Please specify.**[2]

  - (*display optional free text*)

- ❏ None of these fully describe me

  *Branching Logic: when "None of these fully describe me" selected, then:*

  **Please specify.**[2]

  - (*display optional free text*)

- Asian (For example: Asian Indian, Chinese, Filipino, Japanese, Korean, Vietnamese, etc.)
  [Original source question text: Asian – Provide details below.]
  *Branching Logic: when "Asian" selected, then select:*
  - ❏ Asian Indian
  - ❏ Cambodian
  - ❏ Chinese
  - ❏ Filipino
  - ❏ Hmong
  - ❏ Japanese
  - ❏ Korean
  - ❏ Pakistani
  - ❏ Vietnamese
  - ❏ None of these fully describe me

    *Branching Logic: when "None of these fully describe me" selected, then:*

    **Please specify.**[2]

    - (*display optional free text*)

❏ Black, African American, or African (For example: African American, Ethiopian, Haitian, Jamaican, Nigerian, Somali, etc.)
[Original source question text: Black or African Am. – Provide details below.]
*Branching Logic: when "Black, African American, or African" selected, then select:*
- ❏ African American
- ❏ Barbadian
- ❏ Caribbean

- ❑ Ethiopian
- ❑ Ghanaian
- ❑ Haitian
- ❑ Jamaican
- ❑ Liberian
- ❑ Nigerian
- ❑ Somali
- ❑ South African
- ❑ None of these fully describe me

  *Branching Logic: when "None of these fully describe me" selected, then:*
  **Please specify.**[2]
  - (*display optional free text*)

❑ Hispanic, Latino, or Spanish (For example: Colombian, Cuban, Dominican, Mexican or Mexican American, Puerto Rican, Salvadoran, etc.)
[Original source question text: Hispanic, Latino, or Spanish – Provide details below.]
*Branching Logic: when "Hispanic, Latino, or Spanish" selected, then select:*

- ❑ Colombian
- ❑ Cuban
- ❑ Dominican
- ❑ Ecuadorian
- ❑ Honduran
- ❑ Mexican or Mexican American
- ❑ Puerto Rican
- ❑ Salvadoran
- ❑ Spanish
- ❑ None of these fully describe me

  *Branching Logic: when "None of these fully describe me" selected, then:*
  **Please specify.**[2]
  - (*display optional free text*)

- Middle Eastern or North African (For example: Algerian, Egyptian, Iranian, Lebanese, Moroccan, Syrian, etc.)
  [Original source question text: Middle Eastern or North African – Provide details below.]
  *Branching Logic: when "Middle Eastern or North African" selected, then select:*

- ❑ Afghan
- ❑ Algerian
- ❑ Egyptian
- ❑ Iranian
- ❑ Iraqi
- ❑ Israeli
- ❑ Lebanese
- ❑ Moroccan

- ❑ Syrian
- ❑ Tunisian
- ❑ None of these fully describe me

  *Branching Logic: when "None of these fully describe me" selected, then:*
  **Please specify.**[2]
  - *(display optional free text)*

- Native Hawaiian or other Pacific Islander (For example: Chamorro, Fijian, Marshallese, Native Hawaiian, Tongan, etc.)
  [Original source question text: Native Hawaiian or other Pacific Islander – Provide details below.]
  *Branching Logic: when "Native Hawaiian or other Pacific Islander" selected, then select:*
  - ❑ Chamorro
  - ❑ Chuukese
  - ❑ Fijian
  - ❑ Marshallese
  - ❑ Native Hawaiian
  - ❑ Palauan
  - ❑ Samoan
  - ❑ Tahitian
  - ❑ Tongan
  - ❑ None of these fully describe me

    *Branching Logic: when "None of these fully describe me" selected, then:*
    **Please specify.**[2]
    - *(display optional free text)*

- White (For example: English, European, French, German, Irish, Italian, Polish, etc.)
  [Original source question text: White – Provide details below.]
  *Branching Logic: when "White" selected, then select:*
  - ❑ Dutch
  - ❑ English
  - ❑ European
  - ❑ French
  - ❑ German
  - ❑ Irish
  - ❑ Italian
  - ❑ Norwegian
  - ❑ Polish
  - ❑ Scottish
  - ❑ Spanish
  - ❑ None of these fully describe me

    *Branching Logic: when "None of these fully describe me" selected, then:*
    **Please specify.**[2]
    - *(display optional free text)*

- ❑ None of these fully describe me
  *Branching Logic: when "None of these fully describe me" selected, then:*
  **Please specify.**[2]
    - (*display optional free text*)
- ❑ Prefer not to answer

**What terms best express how you describe your gender identity? (Check all that apply)**[3]
[Original source question text: What is your current gender identity? (Check all that apply)]

- ❑ Man
- ❑ Woman
- ❑ Non-binary
- ❑ Transgender
- ❑ None of these describe me, and I'd like to consider additional options
- ❑ Prefer not to answer

  *Branching Logic: when "non-binary," "transgender," or "None of these describe me, and I'd like to consider additional options" selected, then:*
  **Are any of these a closer description to your gender identity? (Check all that apply)**[3]
  [Original source question text: Not applicable]
    - ❑ Trans man/Transgender Man/FTM
    - ❑ Trans woman/Transgender Woman/MTF
    - ❑ Genderqueer
    - ❑ Genderfluid
    - ❑ Gender variant
    - ❑ Two-spirit
    - ❑ Questioning or unsure of your gender identity
    - ❑ None of these describe me, and I want to specify
      *Branching Logic: when "None of these fully describe me, and I want to specify" selected, then:*
      **Please specify.**[2]
      (*display optional free text*)

**What was your biological sex assigned at birth?**[3]
[Original source question text: What sex were you assigned at birth, on your original birth certificate?]

- Female
- Male
- Intersex
- None of these describe me
  *Branching Logic: when "None of these fully describe me" selected, then:*
  **Please specify.**[3]
    - (*display optional free text*)
- Prefer not to answer

**Which of the following best represents how you think of yourself? (Check all that apply)**[4]
[Original source question text: Which of the following best represents how you think of yourself?]

- ❑ Gay
- ❑ Lesbian
- ❑ Straight; that is, not gay or lesbian, etc.
- ❑ Bisexual
- ❑ None of these describe me, and I'd like to see additional options

  *Branching Logic: when "none of these describe me, and I'd like to see additional options" selected, then:*

  **Are any of these a closer description of how you think of yourself?**[5]
  [Original source question text: What do you mean by something else?]

  - ☐ Queer
  - ☐ Polysexual, omnisexual, sapiosexual or pansexual
  - ☐ Asexual
  - ☐ Two-spirit
  - ☐ Have not figured out or are in the process of figuring out your sexuality
  - ☐ Mostly straight, but sometimes attracted to people of your own sex
  - ○ Do not think of yourself as having sexuality
  - ○ Do not use labels to identify yourself
  - ○ Don't know the answer
  - ○ No, I mean something else

    *Branching Logic: when "No, I mean something else" selected, then:*

    **Please specify.**[5]

    - (*display optional free text*)
- ❑ Prefer not to answer

**What is the highest grade or year of school you completed?**[5]
[Original source question text: What is the highest grade or year of school you completed?]

- Never attended school or only attended kindergarten
- Grades 1 through 4 (Primary)
- Grades 5 through 8 (Middle school)
- Grades 9 through 11 (Some high school)
- Grade 12 or GED (High school graduate)
- 1 to 3 years after high school (Some college, Associate's degree, or technical school)
- College 4 years or more (College graduate)
- Advanced degree (Master's, Doctorate, etc.)
- Prefer not to answer

**Have you ever served on active duty in the United States Armed forces, either in the regular military or in a National Guard or military reserve unit?**

**Note: Active duty does not include training for the Reserves or National Guard, but DOES include activation, for example, for the Persian Gulf War**[5]
[Original source question text: Have you ever served on active duty in the United States Armed Forces, either in the regular military or in a National Guard or military reserve unit?]

- Yes
- No
- Prefer not to answer

**What is your current marital status?**[5]
[Original source question text: Are you…?]

- Married
- Divorced
- Widowed
- Separated
- Never married
- Living with partner
- Prefer not to answer

---

**The next 2 questions ask about any people who live with you.**

**Not including yourself, how many other people live at home with you?** [5]
[Original source question text: How many members of your household, including yourself, are 18 years of age or older?]

- Free text (Integer value)
  *Branching logic: when any number other than "0" is entered, then:*
  **Think of other people who live with you. How many are under the age of 18 years?**[1]
  [Original source question text: How many children less than 18 years of age live in your household?]
    - Free text (Integer value)

---

**The next questions are about health insurance. Include health insurance obtained through employment or purchased directly as well as government programs like Medicare and Medicaid that provide medical care or help pay medical bills.**

**Are you covered by health insurance or some other kind of health care plan?**[1]
[Original source question text: Are you covered by health insurance or some other kind of health care plan?]

- Yes
  *Branching logic: when "Yes" selected, then:*
  **Are you currently covered by any of the following types of health insurance or health coverage plans? Select all that apply from one group.**[1]

[Original source question text: Is this person CURRENTLY covered by any of the following types of health insurance or health coverage plans? Mark “Yes” or “No” for EACH type of coverage.]

- Insurance purchased directly from an insurance company (by you or another family member)
- Insurance through a current or former employer or union (by you or another family member)
- Medicare, for people 65 and older or people with certain disabilities
- Medicaid, Medical Assistance, or any kind of government-assistance plan for those with low incomes or disability
- TRICARE or other military health care
- Veterans Affairs (VA) (including those who have ever used or enrolled for VA health care)
- Indian Health Service
- Any other type of health insurance or health coverage plan
  *Branching logic: when “Any other type of health insurance or health coverage plan” is selected, then:*
  **Please specify:**[1]
  *(free text)*
- I don't have health insurance, self-pay

- No
- Don’t know
- Prefer not to answer

---

**The next questions ask if you have a disability. Some questions will ask you about more than one disability at a time. Please answer “Yes” if you have any one of them.** *(info button text: In 1990, Congress passed a civil rights law to protect people with disabilities. The name of that law is the ADA (Americans with Disabilities Act). Having a disability means you might have a physical or mental problem. That problem might make it hard to do certain things. You might have a problem with: walking, breathing, learning, reading, communicating, seeing, hearing, or thinking.)*

*Implementation Note: See Appendix A below for additional information on these items have been collected.*

**Are you deaf or do you have serious difficulty hearing?**[10]
[Original source question text: Is this person deaf or does he/she have serious difficulty hearing?]

- Yes
- No
- Prefer not to answer

**Are you blind or do you have serious difficulty seeing, even when wearing glasses?**[10]

[Original source question text: Is this person blind or does he/she have serious difficulty seeing even when wearing glasses?]

- Yes
- No
- Prefer not to answer

**Because of a physical, mental, or emotional condition, do you have serious difficulty concentrating, remembering or making decisions?**[10]
[Original source question text: Because of a physical, mental, or emotional condition, does this person have serious difficulty concentrating, remembering, or making decisions?]

- Yes
- No
- Prefer not to answer

**Do you have serious difficulty walking or climbing stairs?**[10]
[Original source question text: Does this person have serious difficulty walking or climbing stairs?]

- Yes
- No
- Prefer not to answer

**Do you have difficulty dressing or bathing?**[10]
[Original source question text: Does this person have difficulty dressing or bathing?]

- Yes
- No
- Prefer not to answer

**Because of a physical, mental, or emotional condition, do you have difficulty doing errands alone such as visiting doctor's office or shopping?**[10]
[Original source question text: Because of a physical, mental, or emotional condition, does this person have difficulty doing errands alone such as visiting a doctor's office or shopping?]

- Yes
- No
- Prefer not to answer

---

**The next questions are about your job, income, and where you live.**

**What is your current employment status? Please select 1 or more of these categories.**[5]
[Original source question text: Are you currently…?]

- ❏ Employed for wages (part- time or full-time)
- ❏ Self-employed

- ❏ Out of work for 1 year or more
- ❏ Out of work for less than 1 year
- ❏ A homemaker
- ❏ A student
- ❏ Retired
- ❏ Unable to work (disabled)
- ❏ Prefer not to answer

*Branching logic: when "employed for wages or self-employed" selected, then:*
**Sharing where you work may help us learn about how the environment affects health. Sharing your work address is your choice. You can say no and still take part in the program.**

**What is your work street address?**[10]
[Original source question text: Not applicable]

- ○ Enter Address
  *Branching Logic: when "Enter Address" selected, then:*
  **Address Line 1**[10]
  ______________________
  **Address Line 2 (optional)**[10]
  ______________________
  **City**[10]
  ______________________
  **State**[10]
  ______________________
  **Zip code**[10]
  ______________________
  **Country**[10]
  ______________________
- ○ Prefer not to answer

---

**One of the things we're trying to understand is how people's income may affect their use of health services. Household income includes your income plus the income of all family members in your household for the last calendar year. Include all wages and other sources of income.**

**What is your annual household income from all sources?**[5]
[Original source question text: Is your annual household income from all sources-]

- Less than $10,000
- $10,000- $24,999
- $25,000-$34,999
- $35,000-$49,999

- $50,000- $74,999
- $75,000-$99,999
- $100,000- $149,999
- $150,000- $199,999
- $200,000 or more
- Prefer not to answer

**Do you own or rent the place where you live?**[5]
[Original source question text: Do you own or rent your home? (Home is defined as the place where you live most of the time/the majority of the year.)]

- Own
- Rent
- Other arrangement
  *Branching logic: when "Other arrangement" selected, then:*
  **Where are you currently living?**[7]
  [Original source question text: Where are you currently living?]
  - On a college campus
  - With a friend/roommate
  - With family
  - Motel/hotel
  - Hospital, rehabilitation center, drug treatment center, or other temporary institution
  - In a group home, nursing home, or other residential facility
  - Transitional housing
  - Emergency shelter or homeless shelter
  - Anywhere outside (e.g., street, vehicle, abandoned building)
  - Other
    *Branching Logic: when "Other" selected, then:*
    **Please specify.**[7]
    *(free text)*
- Prefer not to answer

**How many years have you lived at your current address?**[8]
[Original source question text: How many years have you lived at your current address?]

- Less than 1 year
- 1-2 years
- 3-5 years
- 6-10 years
- 11-20 years
- More than 20 years

---

**The next question is about stress that you may feel about money.**

**In the past 6 months, have you been worried or concerned about NOT having a place to live?**[9]

[Original source question text: Are you worried or concerned that in the next 2 months you may NOT have stable housing that you own, rent, or stay in as part of a household?]

- Yes
- No

---

**If you have a Social Security Number, sharing it with us may help add extra data to the All of Us database. This extra data may come from places like your health care provider or pharmacy. Sharing your Social Security Number is your choice. You can say no and still take part in the program.**

**What is your Social Security Number?**[6]
[Original source question text: Not applicable]

- Enter Social Security number
  *Branching Logic: when "Enter Social Security Number" selected, then:*
  **Social Security Number**[6]
  *(social security number)*
- Prefer not to answer

---

**To help us stay in touch with you in the future, the last section asks for contact information for family and/or friends. This information is not required in order to participate in the program. All information will be securely stored.**

**The *All of Us* Research Program may contact you periodically to gather additional health related information. In case we cannot contact you, please provide the names, addresses, and telephone numbers of 2 relatives or friends who would know where you could be reached in case we have trouble reaching you. (Please give us the names of persons not currently living in the household)**[1]
[Original source question text: The Centers for Disease Control and Prevention may wish to contact you again to obtain additional health related information. Please give me the names, addresses, and telephone numbers of 2 relatives or friends who would know where you could be reached in case we have trouble reaching you. (Please give me the names of persons not currently living in the household.)]

- **Person 1 First Name**[1]
  ____________________
- **Person 1 Middle Initial**[1]
  ____________________
- **Person 1 Last Name**[1]
  ____________________
- **Person 1 Address 1**[1]
  ____________________

- **Person 1 Address 2**[1]

  ______________________
- **Person 1 City**[1]

  ______________________
- **Person 1 State**[1]

  ______________________
- **Person 1 Zip Code**[1]

  ______________________
- **Person 1 Email Address**[1]

  ______________________
- **Person 1 Phone Number**[1] *(allow none, refused, or don't know)*

  ______________________
- **Relationship to You**[1]
  - Child
  - Friend
  - Parent or Guardian
  - Relative
  - Spouse or Partner
- **Person 2 First Name**[1]

  ______________________
- **Person 2 Middle Initial**[1]

  ______________________
- **Person 2 Last Name**[1]

  ______________________
- **Person 2 Address 1**[1]

  ______________________
- **Person 2 Address 2**[1]

  ______________________
- **Person 2 City**[1]

  ______________________
- **Person 2 State**[1]

  ______________________
- **Person 2 Zip Code**[1]

  ______________________
- **Person 2 Email Address**[1]

  ______________________
- **Person 2 Phone Number**[1] *(allow none, refused, or don't know)*

  ______________________
- **Relationship to You**[1]
  - Child
  - Friend
  - Parent or Guardian
  - Relative
  - Spouse or Partner

**Thank you for completing the Basics survey.**

**The information you have shared may contribute to helping researchers improve the health of generations to come.**

**Sources**

1. National Health and Nutrition Examination Survey (NHANES). Hyattsville, MD: National Center for Health Statistics. Available from: https://wwwn.cdc.gov/nchs/nhanes/continuousnhanes/questionnaires.aspx?BeginYear=2017
   a. Year of Original Source: 2017
   b. Brief Description of Source: The National Health and Nutrition Examination Survey (NHANES) is a program conducted by the National Center for Health Statistics that aims to assess the health and nutritional status of the United States population. Approximately 5,000 participants from different counties across the nation complete the NHANES interview each year. Specific survey components include demographic, socioeconomic, dietary, and health-related questions.
2. Matthews K, Phelan J, Jones N, Konya S, Marks R, Pratt B, et al. 2015 National Content Test: Race and Ethnicity Analysis Report. Washington, D.C.: United States Census Bureau. Available from: https://www.census.gov/programs-surveys/decennial-census/decade/2020/planning-management/plan/final-analysis/2015nct-race-ethnicity-analysis.html
   a. Year of Original Source: 2015
   b. Brief Description of Source: In 2015, in preparation for the 2020 Census, the U.S. Census Bureau tested new methods for capturing more accurate data on race and ethnicity than had been captured in previous census years. In 2018, the Census Bureau decided not to use the detailed versions of these questions.
3. Gender Identity in U.S. Surveillance (GenIUSS). Los Angeles, CA: The Williams Institute. Available from: https://williamsinstitute.law.ucla.edu/publications/geniuss-trans-pop-based-survey/
   a. Year of Original Source: 2014
   b. Brief Description of Source: To address gaps in federally-supported population health surveys in the identification of gender minorities, the Gender Identity in the U.S. Surveillance (GenIUSS) Group, offered recommendations for collecting information about sex assigned at birth and current gender identity. The questions are published in their 2014 report, “Best Practices for Asking Questions to Identify Transgender and Other Gender Minority Respondents on Population-Based Surveys.”
4. National Health Interview Survey (NHIS). Hyattsville, MD: National Center for Health Statistics. Available from: https://www.cdc.gov/nchs/nhis/nhis_questionnaires.htm
   a. Year of Original Source: 2016
   b. Brief Description of Source: The National Health Interview Survey (NHIS) is a program of the CDC’s National Center for Health Statistics. Since 1957, the NHIS has collected data on a variety of socioeconomic characteristics and health topics (e.g., conditions, medications, access to medical care, etc.) every 10-15 years.

5. Behavioral Risk Factor Surveillance System (BRFSS). Atlanta, GA: Centers for Disease Control and Prevention. Available from: https://www.cdc.gov/brfss/questionnaires/index.htm
    a. Year of Original Source: 2016
    b. Brief Description of Source: The Behavioral Risk Factor Surveillance System (BRFSS), established in 1984, is an annual national health-related telephone survey. Each year, data is collected on approximately 400,000 adults across the United States. about health-related risk behaviors, chronic health conditions, and use of preventive services.
6. Developed for use by *All of Us*
    a. Year of Original Source: Not Applicable
    b. Brief Description of Source: Not Applicable
7. National Health Care for the Homeless Council (NHCHC). Nashville, TN: National Health Care for the Homeless Council. Available from: https://nhchc.org/wp-content/uploads/2019/08/ask-code-documenting-homelessness-throughout-the-healthcare-system.pdf
    a. Year of Original Source: 2016
    b. Brief Description of Source: A report from the National Health Care for the Homeless Council issued in October 2016 provides a list of seven questions as examples of ways to ask about housing status in the health care setting.
8. UK Biobank. Stockport, UK: UK Biobank. Available from: https://www.ukbiobank.ac.uk/
    a. Year of Original Source: 2011
    b. Brief Description of Source: UK Biobank recruited 500,000 people across the country aged between 40 and 69 years from 2006 to 2010. These half a million participants agreed to have their health followed so scientists across the globe can help improve the prevention, diagnosis and treatment of a wide range of diseases. Assessments were undertaken in 22 centres in Scotland, England and Wales. There were five parts to the UK Biobank assessment process, which lasted between 2-3 hours. These included: Written consent Touch screen questionnaires i.e. detailed diet recall Face-to-face interview with a study nurse Measurements i.e. hand grip, spirometry and bone density Sample collection of blood, urine and saliva In addition to information collected during the baseline assessment, 100,000 UK Biobank participants have worn a 24-hour activity monitor for a week, 20,000 have undertaken repeat measures, and nearly 50,000 have had their heart, brain and abdomen scanned as part of a major imaging project. UK Biobank has grown exponentially since this initial assessment and has become a powerful research resource. The database, which is regularly augmented with additional data, is globally accessible to approved researchers and scientists undertaking vital research into the most common and life-threatening diseases. anywhere in the world.
9. The Homeless Screener Clinical Reminder (HSCR). Washington DC: U.S. Department of Veteran Affairs. [cited 2022 Nov 15]. Available from: https://www.va.gov/HOMELESS/nchav/resources/prevention/homeless-screener.asp
    a. Year of Original Source: 2012

   b. Brief Description of Source: The VA Homelessness Screening Clinical Reminder was developed in 2012 by the National Center on Homelessness among Veterans for use by the Veterans Health Administration to capture homelessness and risk of homelessness in the VA patient population.

10. American Community Survey (ACS). Washington, D.C.: American Community Survey. Available from: https://www2.census.gov/programs-surveys/acs/methodology/questionnaires/2019/quest19.pdf
    a. Year of Original Source: 2018
    b. The American Community Survey collects long-form census data such as ancestry, citizenship, education, income, disability, employment, etc. on a monthly basis and is aggregated yearly. This survey adds detail that was previously difficult to collect in the decennial census.

# Appendix A

## Life Functioning Survey

*Survey Implementation Note: The six disability items present in the "Basics" survey were added there after initial fielding began. If participants completed the "Basics" survey before these items were added, they were administered a stand-alone survey called "Life Functioning." The "Life Functioning" survey consists of these same six disability questions and only administered to participants who completed the "Basics" survey before the disability questions were added.*

This survey asks questions about your life functioning including disabilities you may have. It takes less than 2 minutes to complete. Please answer each question as honestly as possible. There are no right or wrong answers to any of the questions. We are looking for your own answers, and not what you think your doctors, family, or friends want you to say.

Don't feel like you have to spend a long time on each question. The first answer that comes to you is usually the best one. If you aren't sure how to answer a question, choose the best answer from the options given. To ensure your privacy, your name will be separated from your answers before they are shared with researchers.

These six questions ask if you have a disability. Some questions will ask you about more than one disability at a time. Please answer "Yes" if you have any one of them.

[Optional Informational Pop-up: "In 1990, Congress passed a civil rights law to prohibit discrimination against people with disabilities. The name of that law is the ADA (Americans with Disabilities Act). Having a disability means you might have a physical or mental health condition. That condition might make it difficult to walk, breathe, learn, read, communicate, see, hear, or think."]

---

**Are you deaf or do you have serious difficulty hearing?**[1]
[Original source question text: Are you deaf or do you have serious difficulty hearing?]

- Yes
- No
- Prefer not to answer

**Are you blind or do you have serious difficulty seeing, even when wearing glasses?**[1]
[Original source question text: Are you blind or do you have serious difficulty seeing, even when wearing glasses?]

- Yes
- No
- Prefer not to answer

**Because of a physical, mental, or emotional condition, do you have serious difficulty concentrating, remembering or making decisions?**[1]
[Original source question text: Because of a physical, mental, or emotional condition, do you have serious difficulty concentrating, remembering or making decisions?]

- Yes
- No
- Prefer not to answer

**Do you have serious difficulty walking or climbing stairs?**[1]
[Original source question text: Do you have serious difficulty walking or climbing stairs?]

- Yes
- No
- Prefer not to answer

**Do you have difficulty dressing or bathing?**[1]
[Original source question text: Do you have difficulty dressing or bathing?]

- Yes
- No
- Prefer not to answer

**Because of a physical, mental, or emotional condition, do you have difficulty doing errands alone such as visiting doctor's office or shopping?**[1]
[Original source question text: Because of a physical, mental, or emotional condition, do you have difficulty doing errands alone such as visiting doctor's office or shopping?]

- Yes
- No
- Prefer not to answer

---

Thank you for completing this survey.

Your experiences can help researchers better understand and improve health for *All of Us*.

Sources

1. *All of Us* Basics Survey; Basics items originally derived from: American Community Survey (ACS). Washington, D.C.: American Community Survey. Available from: https://www2.census.gov/programs-surveys/acs/methodology/questionnaires/2019/quest19.pdf
    1. Year of Original Source: 2018
    2. The American Community Survey collects long-form census data such as ancestry, citizenship, education, income, disability, employment, etc. on a monthly basis and is aggregated yearly. This survey adds detail that was previously difficult to collect in the decennial census.

# Social Determinants of Health (SDOH)

Thank you (again) for being a part of the *All of Us* Research Program. Many things can make us sick or keep us healthy. Where you were born or where you live, go to school, or work can affect your health. These are sometimes referred to as social factors, or the "social determinants," of health. Help us learn more by completing this survey about your neighborhood, social life, stress, and feelings about your everyday life. This survey will take less than 10 minutes to complete.

Please answer each question as honestly as possible. It is important that you answer as many questions as you can. We are looking for your own answers, and not what you think your doctors, family, or friends want you to say.

Don't feel like you have to spend a long time over each question. The first answer that comes to you is usually the best one. If you aren't sure how to answer a question, choose the best answer from the options given. Some of the questions may be sensitive. You can choose not to answer.

---

**The neighborhood where you live may play a role in a variety of health outcomes. Sharing details about your neighborhood may help researchers better understand the relationship between a person's neighborhood and their overall well-being.**

**The following statements describe what your neighborhood might be like. Tell us how much you agree or disagree.**

**People around here are willing to help their neighbors.**[1]
[Original Source Question Text: People around here are willing to help their neighbors.]
- Strongly agree
- Agree
- Neutral (neither agree nor disagree)
- Disagree
- Strongly disagree

**People in my neighborhood generally get along with each other.**[1]
[Original Source Question Text: People in my neighborhood generally get along with each other.]
- Strongly agree
- Agree

- Neutral (neither agree nor disagree)
- Disagree
- Strongly disagree

**People in my neighborhood can be trusted.**[1]
[Original Source Question Text: People in my neighborhood can be trusted.]
- Strongly agree
- Agree
- Neutral (neither agree nor disagree)
- Disagree
- Strongly disagree

**People in my neighborhood share the same values.**[1]
[Original Source Question Text: People in my neighborhood share the same values.]
- Strongly agree
- Agree
- Neutral (neither agree nor disagree)
- Disagree
- Strongly disagree

**There is a lot of graffiti in my neighborhood.**[2]
[Original Source Question Text: There is a lot of graffiti in my neighborhood.]
- Strongly disagree
- Disagree
- Agree
- Strongly agree

**My neighborhood is noisy.**[2]
[Original Source Question Text: My neighborhood is noisy.]
- Strongly disagree
- Disagree
- Agree
- Strongly agree

**Vandalism is common in my neighborhood.**[2]
[Original Source Question Text: Vandalism is common in my neighborhood.]
- Strongly disagree
- Disagree

- Agree
- Strongly agree

**There are lot of abandoned buildings in my neighborhood.**[2]
[Original Source Question Text: There are a lot of abandoned buildings in my neighborhood.]
- Strongly disagree
- Disagree
- Agree
- Strongly agree

**My neighborhood is clean.**[2]
[Original Source Question Text: My neighborhood is clean.]
- Strongly disagree
- Disagree
- Agree
- Strongly agree

**People in my neighborhood take good care of their houses and apartments.**[2]
[Original Source Question Text: People in my neighborhood take good care of their houses and apartments.]
- Strongly disagree
- Disagree
- Agree
- Strongly agree

**There are too many people hanging around on the streets near my home.**[2]
[Original Source Question Text: There are too many people hanging around on the streets near my home.]
- Strongly disagree
- Disagree
- Agree
- Strongly agree

**There is a lot of crime in my neighborhood.**[2]
[Original Source Question Text: There is a lot of crime in my neighborhood.]
- Strongly disagree
- Disagree
- Agree

- Strongly agree

**There is too much drug use in my neighborhood.**[2]
[Original Source Question Text: There is too much drug use in my neighborhood.]
- Strongly disagree
- Disagree
- Agree
- Strongly agree

**There is too much alcohol use in my neighborhood.**[2]
[Original Source Question Text: There is too much alcohol use in my neighborhood.]
- Strongly disagree
- Disagree
- Agree
- Strongly agree

**I'm always having trouble with my neighbors.**[2]
[Original Source Question Text: I'm always having trouble with my neighbors.]
- Strongly disagree
- Disagree
- Agree
- Strongly agree

**In my neighborhood, people watch out for each other.**[2]
[Original Source Question Text: In my neighborhood, people watch out for each other.]
- Strongly disagree
- Disagree
- Agree
- Strongly agree

**My neighborhood is safe.**[2]
[Original Source Question Text: My neighborhood is safe.]
- Strongly disagree
- Disagree
- Agree
- Strongly agree

**The next questions ask about what is in your neighborhood. Think about the area around your home that you can walk to in 10–15 minutes.**

**What is the main type of housing in your neighborhood?**[3]
[Original Source Question Text: What is the main type of housing in your neighborhood?]
- Detached single-family housing
- Townhouses, row house, apartments, or condos of 2-3 stories
- Mix of single-family residences and townhouses, row houses, apartments or condos
- Apartments or condos of 4-12 stories
- Apartments or condos of more than 12 stories
- Don't know/Not sure

**Many shops, stores, markets or other places to buy things I need are within easy walking distance of my home. Would you say that you…**[3]
[Original Source Question Text: Many shops, stores, markets or other places to buy things I need are within easy walking distance of my home. Would you say that you…]
- Strongly disagree
- Somewhat disagree
- Somewhat agree
- Strongly agree
- Don't know/Not sure

**It is within a 10–15 minutes walk to a transit stop (such as bus, train, trolley, or tram) from my home. Would you say that you…**[3]
[Original Source Question Text: It is within a 10-15 minute walk to a transit stop (such as bus, train, trolley, or tram) from my home. Would you say that you…]
- Strongly disagree
- Somewhat disagree
- Somewhat agree
- Strongly agree
- Don't know/Not sure

**There are sidewalks on most of the streets in my neighborhood. Would you say that you…**[3]
[Original Source Question Text: There are sidewalks on most of the streets in my neighborhood. Would you say that you…]
- Strongly disagree

- Somewhat disagree
- Somewhat agree
- Strongly agree
- Does not apply to my neighborhood
- Don't know/Not sure

**There are facilities to bicycle in or near my neighborhood, such as special lanes, separate paths or trails, shared use paths for cycles and pedestrians. Would you say that you…**[3]
[Original Source Question Text: There are facilities to bicycle in or near my neighborhood, such as special lanes, separate paths or trails, shared use paths for cycles and pedestrians. Would you say that you…]

- Strongly disagree
- Somewhat disagree
- Somewhat agree
- Strongly agree
- Does not apply to my neighborhood
- Don't know/Not sure

**My neighborhood has several free or low-cost recreation facilities, such as parks, walking trails, bike paths, recreation centers, playgrounds, public swimming pools, etc. Would you say that you…**[3]
[Original Source Question Text: My neighborhood has several free or low-cost recreation facilities, such as parks, walking trails, bike paths, recreation centers, playgrounds, public swimming pools, etc. Would you say that you…]

- Strongly disagree
- Somewhat disagree
- Somewhat agree
- Strongly agree
- Don't know/Not sure

**The crime rate in my neighborhood makes it unsafe to go on walks at night. Would you say that you…**[3]
[Original Source Question Text: The crime rate in my neighborhood makes it unsafe to go on walks at night. Would you say that you…]

- Strongly disagree
- Somewhat disagree
- Somewhat agree
- Strongly agree

- Don't know/Not sure

**The crime rate in my neighborhood makes it unsafe to go on walks during the day. Would you say that you…**[3]
[Original Source Question Text: The crime rate in my neighborhood makes it unsafe to go on walks during the day. Would you say that you…]

- Strongly disagree
- Somewhat disagree
- Somewhat agree
- Strongly agree
- Don't know/Not sure

---

**Supportive relationships may play a key role in helping people live healthy lives. Answering the following questions may help researchers learn more about the potential health benefits of supportive relationships.**

**People sometimes look to others for friendship, help, or other types of support. Choose the answer that best describes how often you can find support if you need it?**

---

**Someone to help you if you were confined to bed**[4]
[Original Source Question Text: Someone to help you if you were confined to bed]

- None of the time
- A little of the time
- Some of the time
- Most of the time
- All of the time

**Someone to take you to the doctor if you need it**[4]
[Original Source Question Text: Someone to take you to the doctor if you needed it]

- None of the time
- A little of the time
- Some of the time
- Most of the time
- All of the time

**Someone to prepare your meals if you were unable to do it yourself**[4]

[Original Source Question Text: Someone to prepare your meals if you were unable to do it yourself]

- None of the time
- A little of the time
- Some of the time
- Most of the time
- All of the time

**Someone to help with daily chores if you were sick**[4]

[Original Source Question Text: Someone to help with daily chores if you were sick]

- None of the time
- A little of the time
- Some of the time
- Most of the time
- All of the time

**Someone to have a good time with**[4]

[Original Source Question Text: Someone to have a good time with]

- None of the time
- A little of the time
- Some of the time
- Most of the time
- All of the time

**Someone to turn to for suggestions about how to deal with a personal problem**[4]

[Original Source Question Text: Someone to turn to for suggestions about how to deal with a personal problem]

- None of the time
- A little of the time
- Some of the time
- Most of the time
- All of the time

**Someone who understands your problems**[4]

[Original Source Question Text: Someone who understands your problems]

- None of the time
- A little of the time
- Some of the time

- Most of the time
- All of the time

**Someone to love and make you feel wanted**[4]
[Original Source Question Text: Someone to love and make you feel wanted]

- None of the time
- A little of the time
- Some of the time
- Most of the time
- All of the time

---

**We would like to ask you some questions about your relationships with others. Choose the answer that is true for you.**

---

**I lack companionship**[5]
[Original Source Question Text: I lack companionship]

- Never
- Rarely
- Sometimes
- Often

**There is no one I can turn to**[5]
[Original Source Question Text: There is no one I can turn to]

- Never
- Rarely
- Sometimes
- Often

**I am an outgoing person**[5]
[Original Source Question Text: I am an outgoing person]

- Never
- Rarely
- Sometimes
- Often

**I feel left out**[5]

[Original Source Question Text: I feel left out]

- Never
- Rarely
- Sometimes
- Often

**I feel isolated from others**[5]

[Original Source Question Text: I feel isolated from others]

- Never
- Rarely
- Sometimes
- Often

**I can find companionship when I want it**[5]

[Original Source Question Text: I can find companionship when I want it]

- Never
- Rarely
- Sometimes
- Often

**I am unhappy being so withdrawn**[5]

[Original Source Question Text: I am unhappy being so withdrawn]

- Never
- Rarely
- Sometimes
- Often

**People are around me but not with me**[5]

[Original Source Question Text: People are around me but not with me]

- Never
- Rarely
- Sometimes
- Often

---

**People who experience discrimination may be at higher risk of negative health outcomes. Sharing your experiences with discrimination in everyday life and in healthcare settings may**

**help researchers learn how to reduce negative health outcomes associated with discrimination.**

**The next statements describe how others may treat you. In your day-to-day life, how often do any of these happen to you?**

---

**You are treated with less courtesy than other people are.**[6]
[Original Source Question Text: You are treated with less courtesy than other people are]

- Almost every day
- At least once a week
- A few times a month
- A few times a year
- Less than once a year
- Never

**You are treated with less respect than other people are.**[6]
[Original Source Question Text: You are treated with less respect than other people are]

- Almost every day
- At least once a week
- A few times a month
- A few times a year
- Less than once a year
- Never

**You receive poorer service than other people at restaurants or stores.**[6]
[Original Source Question Text: You receive poorer service than other people at restaurants or stores.]

- Almost every day
- At least once a week
- A few times a month
- A few times a year
- Less than once a year
- Never

**People act as if they think you are not smart.**[6]
[Original Source Question Text: People act as if they think you are not smart.]

- Almost every day

- At least once a week
- A few times a month
- A few times a year
- Less than once a year
- Never

**People act as if they are afraid of you.**[6]
[Original Source Question Text: People act as if they are afraid of you.]

- Almost every day
- At least once a week
- A few times a month
- A few times a year
- Less than once a year
- Never

**People act as if they think you are dishonest.**[6]
[Original Source Question Text: People act as if they think you are dishonest.]

- Almost every day
- At least once a week
- A few times a month
- A few times a year
- Less than once a year
- Never

**People act as if they're better than you are.**[6]
[Original Source Question Text: People act as if they're better than you are.]

- Almost every day
- At least once a week
- A few times a month
- A few times a year
- Less than once a year
- Never

**You are called names or insulted.**[6]
[Original Source Question Text: You are called names or insulted.]

- Almost every day
- At least once a week
- A few times a month

- A few times a year
- Less than once a year
- Never

**You are threatened or harassed.**[6]
[Original Source Question Text: You are threatened or harassed.]
- Almost every day
- At least once a week
- A few times a month
- A few times a year
- Less than once a year
- Never

**What do you think is the main reason for these experiences?**[6]
[Original Source Question Text: What do you think is the main reason for these experiences? Select all that apply.]
- Your Ancestry or National Origins
- Your Gender
- Your Race
- Your Age
- Your Religion
- Your Height
- Your Weight
- Some Other Aspect of Your Physical Appearance
- Your Sexual Orientation
- Your Education or Income Level
- Other (specify)

  *Branching logic: when "Other (specify)" selected, then:*

  **Please specify.**[14]

  _______________

---

**The next statements describe how others may treat you. How often do any of these happen to you when you go to a doctor's office or other health care provider?**

---

**You are treated with less courtesy than other people.**[7]

[Original Source Question Text: You are treated with less courtesy than other people are]
- Never
- Rarely
- Sometimes
- Most of the time
- Always

**You are treated with less respect than other people.**[7]
[Original Source Question Text: You are treated with less respect than other people are]
- Never
- Rarely
- Sometimes
- Most of the time
- Always

**You receive poorer service than others.**[7]
[Original Source Question Text: You receive poorer service than others.]
- Never
- Rarely
- Sometimes
- Most of the time
- Always

**A doctor or nurse acts if he or she thinks you are not smart.**[7]
[Original Source Question Text: A doctor or nurse acts if he or she thinks you are not smart.]
- Never
- Rarely
- Sometimes
- Most of the time
- Always

**A doctor or nurse acts as if he or she is afraid of you.**[7]
[Original Source Question Text: A doctor or nurse acts as if he or she is afraid of you.]
- Never
- Rarely
- Sometimes
- Most of the time
- Always

**A doctor or nurse acts as if he or she is better than you.**[7]
[Original Source Question Text: A doctor or nurse acts as if he or she is better than you.]

- Never
- Rarely
- Sometimes
- Most of the time
- Always

**You feel like a doctor or nurse is not listening to what you were saying.**[7]
[Original Source Question Text: You feel like a doctor or nurse is not listening to what you were saying.]

- Never
- Rarely
- Sometimes
- Most of the time
- Always

---

**The next set of questions asks about food and housing.**

**Lower food and housing security may increase the risk of negative health outcomes. Sharing your experiences about you or your family on this topic may help researchers learn how food and housing security influence health and well-being.**

---

**Within the past 12 months, we worried whether our food would run out before we got money to buy more.**[8]
[Original Source Question Text: Within the past 12 months, we worried whether our food would run out before we got money to buy more.]

- Often true
- Sometimes true
- Never true

**Within the past 12 months, the food we bought just didn't last and we didn't have money to get more.**[8]
[Original Source Question Text: Within the past 12 months, the food we bought just didn't last and we didn't have money to get more.]

- Often true

- Sometimes true
- Never true

**In the last 12 months, how many times have you or your family moved from one home to another?**
**Number of moves in past 12 months.**[9]
[Original Source Question Text: In the last 12 months, how many times have you or your family moved from one home to another?]
____________ (integer value)

**Think about the place you live. Do you have problems with any of the following (check all that apply)?**[10]
[Original Source Question Text: Think about the place you live. Do you have problems with any of the following (check all that apply)?]

- Bug infestation
- Mold
- Lead paint or pipes
- Inadequate heat
- Oven or stove not working
- No or not working smoke detector
- Water leaks
- None of the above

---

**The amount of stress you feel can lead to negative health outcomes. Sharing your experiences may help researchers identify common triggers that induce stress in individuals and ways to provide support for individuals and communities.**

**The next questions ask you about your feelings and thoughts during the last month. Please choose how often you felt or thought a certain way.**

---

**In the last month, how often have you been upset because of something that happened unexpectedly?**[11]
[Original Source Question Text: In the last month, how often have you been upset because of something that happened unexpectedly?]

- Never
- Almost Never

- Sometimes
- Fairly Often
- Very Often

**In the last month, how often have you felt that you were unable to control the important things in your life?**[11]
[Original Source Question Text: In the last month, how often have you felt that you were unable to control the important things in your life?]

- Never
- Almost Never
- Sometimes
- Fairly Often
- Very Often

**In the last month, how often have you felt nervous and "stressed"?**[11]
[Original Source Question Text: In the last month, how often have you felt nervous and "stressed"?]

- Never
- Almost Never
- Sometimes
- Fairly Often
- Very Often

**In the last month, how often have you felt confident about your ability to handle your personal problems?**[11]
[Original Source Question Text: In the last month, how often have you felt confident about your ability to handle your personal problems?]

- Never
- Almost Never
- Sometimes
- Fairly Often
- Very Often

**In the last month, how often have you felt that things were going your way?**[11]
[Original Source Question Text: In the last month, how often have you felt that things were going your way?]

- Never
- Almost Never

- Sometimes
- Fairly Often
- Very Often

**In the last month, how often have you found that you could not cope with all the things that you had to do?**[11]
[Original Source Question Text: In the last month, how often have you found that you could not cope with all the things that you had to do?]

- Never
- Almost Never
- Sometimes
- Fairly Often
- Very Often

**In the last month, how often have you been able to control irritations in your life?**[11]
[Original Source Question Text: In the last month, how often have you been able to control irritations in your life?]

- Never
- Almost Never
- Sometimes
- Fairly Often
- Very Often

**In the last month, how often have you felt that you were on top of things?**[11]
[Original Source Question Text: In the last month, how often have you felt that you were on top of things?]

- Never
- Almost Never
- Sometimes
- Fairly Often
- Very Often

**In the last month, how often have you been angered because of things that were outside of your control?**[11]
[Original Source Question Text: In the last month, how often have you been angered because of things that were outside of your control?]

- Never
- Almost never

- Sometimes
- Fairly often
- Very often

**In the last month, how often have you felt difficulties were piling up so high that you could not overcome them?**[11]
[Original Source Question Text: In the last month, how often have you felt difficulties were piling up so high that you could not overcome them?]

- Never
- Almost Never
- Sometimes
- Fairly Often
- Very Often

---

**Elements of religion and spirituality can affect health outcomes. Sharing your beliefs and experiences may help researchers better understand the effects of religion and spirituality on health and well-being.**

**The next questions ask about your spiritual life. Some questions use the word "God." If it makes you more comfortable, you can replace that word with whatever you believe is spiritual, holy, or divine when answering.**

**How often do you experience the following:**

---

**I feel God's (or a higher power's) presence**[12]
[Original Source Question Text: I feel God's presence]

- Many times a day
- Every day
- Most days
- Some days
- Once in a while
- Never or almost never
- I do not believe in God (or a higher power)

**I find strength and comfort in my religion**[12]

[Original Source Question Text: I find strength and comfort in my religion]

- Many times a day
- Every day
- Most days
- Some days
- Once in a while
- Never or almost never
- I am not religious

**I feel deep inner peace or harmony**[12]

[Original Source Question Text: I feel deep inner peace or harmony]

- Many times a day
- Every day
- Most days
- Some days
- Once in a while
- Never or almost never

**I desire to be closer to or in union with God (or a higher power)**[12]

[Original Source Question Text: I desire to be closer to or in union with God]

- Many times a day
- Every day
- Most days
- Some days
- Once in a while
- Never or almost never
- I do not believe in God (or a higher power)

**I feel God's (or a higher power's) love for me, directly or through others**[12]

[Original Source Question Text: I feel God's love for me, directly or through others]

- Many times a day
- Every day
- Most days
- Some days
- Once in a while
- Never or almost never
- I do not believe in God (or a higher power)

**I am spiritually touched by the beauty of creation**[12]
[Original Source Question Text: I am spiritually touched by the beauty of creation]

- Many times a day
- Every day
- Most days
- Some days
- Once in a while
- Never or almost never

**How often do you go to religious meetings or services?**[13]
[Original Source Question Text: How often do you go to religious meetings or services?]

- More than once a week
- Once a week
- 1 to 3 times per month
- Less than once per month
- Never (or almost never)
- I am not religious

---

**Having a preferred language that is not English can be a barrier to health care access, use of health care services, and understanding health-related information. Sharing your English-speaking abilities with us can help researchers understand the relationship between speaking English and health outcomes.**

**The following question asks if you speak any languages other than English at home.**

---

**Do you speak a language other than English at home?**[14]
[Original Source Question Text: Does this person speak a language other than English at home?]

- Yes

  *Branching logic: when "Yes" selected, then:*

  **Since you speak a language other than English at home, we are interested in your own thoughts about how well you speak English. Would you say you speak English...**[14]

  [Original Source Question Text: Since you speak a language other than English at home, we are interested in your own opinion of how well you speak English. Would you say you speak English…]

  - Very well
  - Well

- Not well
- Not at all
- Prefer not to answer
- Don’t know

- No
- Prefer not to answer

**Sources:**

1. Bateman LB, Fouad MN, Hawk B, Osborne T, Bae S, Eady S, Thompson J, Brantley W, Crawford L, Heider L, Schoenberger YM. Examining Neighborhood Social Cohesion in the Context of Community-based Participatory Research: Descriptive Findings from an Academic-Community Partnership. Ethn Dis. 2017 Nov 9;27(Suppl 1):329-336. doi: 10.18865/ed.27.S1.329. PMID: 29158658; PMCID: PMC5684777.
    a. Year of Original Source: 2016
    b. Brief Description of Source: The Social Cohesion Neighborhood Scale was developed in partnership between academic researchers and local neighborhood groups to assess perceptions of neighborhood cohesion in the Birmingham, Alabama region. The scale was originally included in a survey administered to 90 neighborhood residents in 2016.
2. Ross CE, Mirowsky J. Disorder and Decay: The Concept and Measurement of Perceived Neighborhood Disorder. Urban Affairs Review. 1999 Jan 1;34(3):412–32.
    a. Year of Original Source: 1995
    b. Brief Description of Source: The Ross-Mirowsky Perceived Neighborhood Disorder Scale was developed to assess the relationship between perceived neighborhood order and disorder. The scale was originally part of the 1995 Survey of Community, Crime and Health, which was administered to select Illinois-based households.
3. Bauman A, Bull F, Chey T, Craig CL, Ainsworth BE, Sallis JF, et al. The International Prevalence Study on Physical Activity: results from 20 countries. Int J Behav Nutr Phys Act. 2009 Mar 31;6:21.
    a. Year of Original Source: 2009
    b. Brief Description of Source: The International Physical Activity Questionnaire (IPAQ) was developed as a tool for comparing physical activity prevalence across the international community. for international surveillance. The questionnaire was originally included in a study of 20 countries from 2002-2004 and administered to over 52,000 participants.
4. Social Support Survey. Santa Monica, CA: RAND Corporation. Available from: https://www.rand.org/health-care/surveys_tools/mos/social-support.html
    a. Year of Original Source: 1993
    b. Brief Description of Source: The Social Support Survey instrument was developed for the two-year  Medical Outcomes Study (MOS), a two-year study of patients with chronic conditions.
5. Hays RD, DiMatteo MR. A short-form measure of loneliness. J Pers Assess. 1987 Spring;51(1):69-81. PMID: 3572711.
    a. Year of Original Source: 1987
    b. Brief Description of Source: The revised 1987 version of the short-form UCLA Loneliness Scale (ULS-8) was developed through factor analysis of the original

20-item, which was released in 1978 to measure one's feelings of loneliness and social connection.

6. Williams DR, Yan Yu, Jackson JS, Anderson NB. Racial Differences in Physical and Mental Health: Socio-economic Status, Stress and Discrimination. J Health Psychol. 1997 Jul;2(3):335–51.
    a. Year of Original Source: 1997
    b. Brief Description of Source: The Everyday Discrimination Scale was developed to assess perceived discrimination in everyday societal life.
7. Peek ME, Nunez-Smith M, Drum M, Lewis TT. Adapting the everyday discrimination scale to medical settings: reliability and validity testing in a sample of African American patients. Ethn Dis. 2011 Autumn;21(4):502-9. PMID: 22428358; PMCID: PMC3350778.
    a. Year of Original Source: 2011
    b. Brief Description of Source: A modified version of the Everyday Discrimination Scale was adapted to measure perceived discrimination within the health care setting. The adapted version was originally deployed to over 70 participants in a Chicago-based academic medical care center.
8. The Hunger Vital Sign™. Boston, MA: Children's HealthWatch. Available from: https://childrenshealthwatch.org/public-policy/hunger-vital-sign/
    a. Year of Original Source: 2010
    b. Brief Description of Source: The Hunger Vital SignTM is a 2-question screening tool developed to identify households at risk of food insecurity. It is based on the U.S. Household Food Security Survey.
9. Manchanda R, Gottlieb L. Upstream Risks Screening Tool and Guide V2.6. Los Angeles, CA: HealthBegins. Available from: https://www.aamc.org/system/files/c/2/442878-chahandout1.pdf
    a. Year of Original Source: 2015
    b. Brief Description of Source: The Health Begins Upstream Risks Screening Tool was designed to assess elements of social determinants of health across 5 domains (economic stability, education, social & community context, neighborhood & physical environment, and food).
10. Accountable Health Communities Model [Internet]. Baltimore, MD: U.S. Centers for Medicare & Medicaid Services. Available from: https://innovation.cms.gov/innovation-models/ahcm
    a. Year of Original Source: 2017
    b. Brief Description of Source: The Accountable Health Communities Model Health-Related Social Needs screening tool was created to identify unmet health-related social needs including housing and food insecurity.
11. Cohen S, Kamarck T, Mermelstein R. A global measure of perceived stress. J Health Soc Behav. 1983 Dec;24(4):385-96. PMID: 6668417.
    a. Year of Original Source: 1983

b. Brief Description of Source: The Perceived Stress Scale is a widely-used instrument for assessing one's perceived levels of stress specific to life events within a one month timeframe.

12. Daily Spiritual Experience Scale: Home. Daily Spiritual Experience Scale. Available from: https://www.dsescale.org/
    a. Year of Original Source: 1997
    b. Brief Description of Source: The Brief Multidimensional Measure of Religiousness/Spirituality – Daily Spiritual Experiences Scale Short Form, originally released in 1997, includes six items meant to measure the impact of religion and spirituality on everyday life.
13. Nurses' Health Study: Questionnaires. Cambridge, MA: Nurses' Health Study. Available from: https://nurseshealthstudy.org/participants/questionnaires
    a. Year of Original Source: 1992
    b. Brief Description of Source: The Nurses' Health Study, led by researchers at Harvard University, is a study meant to assess and understand risk factors for major chronic diseases in women. Questionnaire-based methods have been developed to assess a variety of factors including diet and physical activity.
14. California Health Interview Survey. Los Angeles, CA: UCLA Center for Health Policy Research. Available from: http://healthpolicy.ucla.edu/chis/design/Pages/questionnairesEnglish.aspx
    a. Year of Original Source: 2021
    b. Brief Description of Source: The California Health Interview Survey (CHIS) is conducted on a continuous basis by researchers at the UCLA Center for Health Policy Research. Originally launched in 2001, it is the largest state-based phone and email administered survey in the U.S. and covers items related to a variety of health topics.

# Overall Health

This survey asks questions about your overall health. Your privacy is very important to us. Your answers will only be shared with approved researchers after we have removed your name.

It takes about 5-10 minutes to answer these questions. Please answer each question as honestly as possible. There are no right or wrong answers to any of the questions. It is important that you answer as many questions as you can. We are looking for your own answers, and not what you think your doctors, family, or friends want you to say.

Don't feel like you have to spend a long time over each question. The first answer that comes to you is usually the best one. If you aren't sure how to answer a question, choose the best answer from the options given.

**How confident are you filling out medical forms by yourself?**[1]
[Original Source Question Text: How confident are you filling out medical forms by yourself?]
- Extremely
- Quite a bit
- Somewhat
- A little bit
- Not at all

**How often do you have someone help you read health-related materials?**[1]
[Original Source Question Text: How often do you have someone help you read hospital materials?]
- Always
- Often
- Sometimes
- Occasionally
- Never

**How often do you have problems learning about your medical condition because of difficulty understanding written information?**[1]
[Original Source Question Text: How often do you have problems learning about your medical condition because of difficulty understanding written information?]
- Always
- Often
- Sometimes
- Occasionally
- Never

**The next 10 questions ask you how you feel about your health and daily activities.**

**In general, would you say your health is:**[2]
[Original Source Question Text: In general, would you say your health is:]
- Excellent
- Very Good
- Good
- Fair
- Poor

**In general, would you say your quality of life is:**[2]
[Original Source Question Text: In general, would you say your quality of life is:]
- Excellent
- Very Good
- Good
- Fair
- Poor

**In general, how would you rate your physical health?**[2]
[Original Source Question Text: In general, how would you rate your physical health?]
- Excellent
- Very Good
- Good
- Fair
- Poor

**In general, how would you rate your mental health, including your mood and your ability to think?**[2]
[Original Source Question Text: In general, how would you rate your mental health, including your mood and your ability to think?]
- Excellent
- Very Good
- Good
- Fair
- Poor

**In general, how would you rate your satisfaction with your social activities and relationships?**[2]
[Original Source Question Text: In general, how would you rate your satisfaction with your social activities and relationships?]

- Excellent
- Very Good
- Good
- Fair
- Poor

**To what extent are you able to carry out your everyday physical activities such as walking, climbing stairs, carrying groceries, or moving a chair?**[2]
[Original Source Question Text: To what extent are you able to carry out your everyday physical activities such as walking, climbing stairs, carrying groceries, or moving a chair?]

- Completely
- Mostly
- Moderately
- A little
- Not at all

**In the past 7 days, how would you rate your pain on average?**[2]
[Original Source Question Text: In the past 7 days, how would you rate your pain on average?]

- 0 (No pain)
- 1
- 2
- 3
- 4
- 5
- 6
- 7
- 8
- 9
- 10 (Worst pain imaginable)

**In the past 7 days, how would you rate your fatigue?**[2]
[Original Source Question Text: In the past 7 days, how would you rate your fatigue on average?]

- None
- Mild
- Moderate
- Severe
- Very Severe

**In general, please rate how well you carry out your usual social roles. (This includes activities at home, at work and in your community, and responsibilities as a parent, child,**

**spouse, employee, friend, etc.)**[2]
[Original Source Question Text: In general, please rate how well you carry out your usual social activities and roles. (This includes activities at home, at work and in your community, and responsibilities as a parent, child, spouse, employee, friend, etc.)]

- Excellent
- Very Good
- Good
- Fair
- Poor

**In the past 7 days, how often have you been bothered by emotional problems such as feeling anxious, depressed or irritable?**[2]
[Original Source Question Text: In the past 7 days, how often have you been bothered by emotional problems such as feeling anxious, depressed, or irritable?]

- Never
- Rarely
- Sometimes
- Often
- Always

---

**The next few questions are about women's health issues. Women's health is very important when considering your overall health. Answering these questions will help us get a more complete picture of your total health.**

*Branching Logic: These questions will only be asked if in The Basics the questions about sex at birth were answered "Female," "intersex," or "Please specify."*

**Have your menstrual periods stopped permanently?**[3]
[Original Source Question Text: Have your menstrual periods stopped permanently?]

- No
- Yes, I have no menstrual periods
- Yes, but I have periods induced by hormones
- Not sure
- Prefer not to answer

  *Branching Logic: when "No" or "Not sure" selected for "Have your menstrual periods stopped permanently?", then:*

  **Are you currently pregnant?**[3]

  [Original Source Question Text: Are you currently pregnant?]

  - Yes
  - No

- ○ Not sure
- ○ Prefer not to answer

*Branching Logic: when "Yes, I have no menstrual periods" or "Yes, but I have periods induced by hormones" selected for "Have your menstrual periods stopped permanently?", then:*

**Why did your periods stop?**[3]
[Original Source Question Text: Why did your periods stop?]

- ○ Natural menopause (change of life)
- ○ Surgery (a hysterectomy to remove your uterus and/or an oophorectomy to remove your ovaries)
- ○ Endometrial ablation (removal of the lining of the uterus)
- ○ Medication, chemotherapy, or radiation
- ○ Other
- ○ Not sure
- ○ Prefer not to answer

*Branching Logic: when "Yes, I have no menstrual periods," "Yes, but I have periods induced by hormones," or "Prefer not to answer" selected for "Have your menstrual periods stopped permanently?", then:*

**Have you ever had a hysterectomy (that is, surgery to remove your uterus or womb)?**[3]
[Original Source Question Text: Have you ever had a hysterectomy (that is, surgery to remove your uterus or womb)?]

- No
- Yes
  *Branching Logic: when "Yes" selected, then:*
  **Age of surgery:**[3]
  ___________ (age in years)
- Not sure
- Prefer not to answer
  *Branching Logic: when "No," "Not Sure," or "Prefer not to answer" selected, then:*
  **Have you ever had an ovary removed?**[3]
  [Original Source Question Text: Have you ever had an ovary removed?]
  - ○ No
  - ○ Yes, but only one ovary or part of one ovary
  - ○ Yes, both ovaries
  - ○ Yes, but don't know whether one or both ovaries
  - ○ Not sure
  - ○ Prefer not to answer
    *Branching Logic: when "Yes, but only one ovary or part of one ovary," "Yes, both ovaries," or "Yes, but don't know whether one or both ovaries" selected, then:*
    **Age of surgery:**[3]
    ___________ (age in years)

**A blood sample may be drawn as part of this study. Some conditions or prior procedures, such as a bone marrow transplant, may cause problems in using your blood sample for research. In addition, some information about you, such as your travel history, may not generally be in your medical record but may still be helpful to researchers. Traveling outside the country may increase your risk of coming into contact with certain infections not commonly present in this country. The following questions are asked to address two of these situations.**

**Have you had a transplant of any type?**[4]
[Original Source Question Text: Had a transplant such as organ, tissue, or bone marrow?]

- Yes
  *Branching Logic: when "Yes" selected, then:*
  **If yes, please check all that apply**[4]
  - ❑ Heart
    *Branching Logic: when "Heart" selected, then:*
    **Date of heart transplant:**[4]
    _______________
  - ❑ Kidney
    *Branching Logic: when "Kidney" selected, then:*
    **Date of kidney transplant:**[4]
    _______________
  - ❑ Liver
    *Branching Logic: when "Liver" selected, then:*
    **Date of liver transplant:**[4]
    _______________
  - ❑ Lung
    *Branching Logic: when "Lung" selected, then:*
    **Date of lung transplant:**[4]
    _______________
  - ❑ Pancreas
    *Branching Logic: when "Pancreas" selected, then:*
    **Date of pancreas transplant:**[4]
    _______________
  - ❑ Intestine
    *Branching Logic: when "Intestine" selected, then:*
    **Date of intestine transplant:**[4]
    _______________
  - ❑ Other organ
    *Branching Logic: when "Other organ" selected, then:*
    **Please specify organ:**[5]
    _________________

- ❑ Cornea
  *Branching Logic: when "Cornea" selected, then:*
  **Date of cornea transplant:**[4]

  ______________

- ❑ Bone
  *Branching Logic: when "Bone" selected, then:*
  **Date of bone transplant:**[4]

  ______________

- ❑ Valve
  *Branching Logic: when "Valve" selected, then:*
  **Date of valve transplant:**[4]

  ______________

- ❑ Skin
  *Branching Logic: when "Skin" selected, then:*
  **Date of skin transplant:**[4]

  ______________

- ❑ Blood Vessels
  *Branching Logic: when "Blood vessels" selected, then:*
  **Date of blood vessel transplant:**[4]

  ______________

- ❑ Other Tissue
  *Branching Logic: when "Other Tissue" selected, then:*
  **Please specify tissue:**[5]

  ________________

  **Date of other tissue transplant:**[5]

  ________________

- No
- Don't know

**Have you traveled outside of the country within the past 6 months?**[4]
[Original Source Question Text: In the past three years, have you: Been outside the United States or Canada?]

- Yes
  *Branching Logic: when "Yes" selected, then:*
  **Where?**[5]

  _________________

  **How long were you there? Enter number of days:**[5]

  _________________
- No

**Your answers will help researchers to better understand health, and advance how to prevent and treat disease.**

**Source Information**

1. Chew LD, Bradley KA, Boyko EJ. Brief questions to identify patients with inadequate health literacy. Fam Med. 2004 Sep;36(8):588-94. PMID: 15343421.
    a. Year of Original Source: 2004
    b. Brief Description of Source: The Brief Health Literacy Screen (BHLS) is a three-item health literacy measure that can be administered in a minute or less. The BHLS, developed in 2004, has been used widely in clinical and research settings.
2. PROMIS Global Health Scale. Evanston, IL: HealthMeasures. Available from: https://www.healthmeasures.net/explore-measurement-systems/promis
    a. Year of Original Source: 2009
    b. Brief Description of Source: The NIH's Patient Reported Outcomes Measurement Information System (PROMIS) Global Health Scale, developed in 2009, is a ten-item self-report measure that assesses overall physical and mental health in adults and children.
3. California Teachers Study. Monrovia, CA: California Teachers Study. Available from: https://www.calteachersstudy.org
    a. Year of Original Source: 1995
    b. Brief Description of Source: Since 1995, the California Teachers Study has investigated the incidence and risk factors of breast cancer among more than 130,000 female California teachers and administrators. The longitudinal cohort study is designed to determine the incidence and potential risk factors for breast and other cancers.
4. Blood Donor History Questionnaires. Bethesda, MD: Association for the Advancement of Blood & Biotherapies. Available from: https://www.aabb.org/news-resources/resources/donor-history-questionnaires/blood-donor-history-questionnaires
    a. Year of Original Source: 1992
    b. Brief Description of Source: The AABB Blood Donor History Questionnaire (DHQ), also referred to as the Uniform Donor History Questionnaire (UDHQ), was developed by the AABB (formerly American Association of Blood Banks) for use by blood centers to meet regulatory requirements for screening potential blood donors. It evaluates prospective donors' history against known blood safety risks following FDA regulations/recommendations and AABB Standards.
5. Developed for use by *All of Us*
    a. Year of Original Source: Not Applicable
    b. Brief Description of Source: Not Applicable

# Lifestyle

This survey asks questions about your use of tobacco, alcohol, and drugs. This is to better understand how these things may affect your overall health. Your privacy is very important to us. Your name will be separated from your answers before they are shared with researchers.

It takes about 5-10 minutes to answer these questions. Please answer each question as honestly as possible. It is important that you answer as many questions as you can. We are looking for your own answers, and not what you think your doctors, family, or friends want you to say.

Don't feel like you have to spend a long time over each question. The first answer that comes to you is usually the best one. If you aren't sure how to answer a question, choose the best answer from the options given.

**Have you smoked at least 100 cigarettes in your entire life? (There are 20 cigarettes in a pack.)?**[1, 2]
[Original Source Question Text: Have/Has you/name smoked at least 100 cigarettes in your/his/her life? Note: 100 Cigarettes = Approximately 5 Packs]

- Yes
  *Branching Logic: when "Yes" selected, then:*
  **Do you now smoke cigarettes every day, some days, or not at all?**[1, 2]
  [Original Source Question Text: Do/Does you/name now smoke cigarettes every day, some days, or not at all?]
    - Every day
    - Some days
    - Not at all
    - Don't know
    - Prefer not to answer

  **How old were you when you first started regular cigarette smoking?**[1, 2]
  [Original Source Question Text: How old where/was you/name when you/he/she first started smoking cigarettes FAIRLY REGULARLY?]
    - Enter response
      *Branching Logic: when "Enter response" selected, then:*
      **Age:**[1, 2]
      __________________
    - Don't know
    - Prefer not to answer

  **In the past, have you ever made a serious attempt to quit smoking? That is, have you stopped smoking for at least one day or longer because you were trying to quit?**[1, 2]

[Original Source Question Text: In the past, have you ever made a serious attempt to quit smoking? That is, have you stopped smoking for at least one day or longer because you were trying to quit?]

- Yes

  *Branching Logic: when "Yes" selected, then:*

  **If you have completely stopped smoking cigarettes, about how old were you when you stopped?**[3]

  [Original Source Question Text: At what age did you last stop smoking cigarettes regularly? (Enter age last stopped smoking)]

  - Enter response

    *Branching Logic: when "Enter response" selected, then:*

    **Age when you stopped smoking:**[3]

    _______________
  - Don't know
  - Prefer not to answer

  **How many years have you or did you smoke cigarettes?**[3]

  [Original Source Question Text: How many years have you or did you smoke cigarettes?]

  - Enter response

    *Branching Logic: when "Enter response" selected, then:*

    **Number of years:**[3]

    _______________
  - Don't Know
  - Prefer not to answer

  **On average, how many cigarettes do you smoke per day now? (There are 20 cigarettes in a pack.)**[1, 2]

  [Original Source Question Text: On the average, about how many cigarettes do you now smoke each day?]

  - Enter response

    *Branching Logic: when "Enter response" selected, then:*

    **Number of cigarettes per day:**[1, 2]

    _______________
  - Don't know
  - Prefer not to answer

  **On average, over the entire time that you smoked, how many cigarettes did you smoke each day? (There are 20 cigarettes in a pack.)**[1, 2]

  [Original Source Question Text: When you last smoked every day, on average how many cigarettes did you smoke each day?

  - Enter response

    *Branching Logic: when "Enter response" selected, then:*

    **Number of cigarettes per day:**[1, 2]

    _______________
  - Don't know

            - Prefer not to answer
    - No
    - Don't know
    - Prefer not to answer

- No
- Don't know
- Prefer not to answer

**Have you ever used an electronic nicotine product, even one or two times? (Electronic nicotine products include e- cigarettes, vape pens, hookah pens, personal vaporizers and mods, e-cigars, e-pipes, and e-hookahs.)**[4]
[Original Source Question Text: Have you ever used e-cigarettes fairly regularly?]

- Yes
  *Branching Logic: when "Yes" selected, then:*
  **Do you now use electronic nicotine products…**[4]
  [Original Source Question Text: Do you now use e-cigarettes…]
    - Every day
    - Some days
    - Not at all
    - Don't know
    - Prefer not to answer

- No
- Don't Know
- Prefer Not To Answer

**Have you ever smoked a traditional cigar, cigarillo, or filtered cigar, even one of two puffs?**[4]
[Original Source Question Text: Have you ever smoked a traditional cigar, even one or two puffs?]

- Yes
  *Branching Logic: when "Yes" selected, then:*
  **Do you now smoke a traditional cigar, cigarillo, or filtered cigar…**[4]
  [Original Source Question Text: [Do you now smoke [[CIGARFILL]s I cigarillos as blunts I filtered cigars as blunts]…]
    - Every day
    - Some days
    - Not at all
    - Don't know
    - Prefer not to answer

- No
- Don't Know

- Prefer Not To Answer

**Have you ever smoked tobacco in a hookah, even one or two puffs?**[4]
[Original Source Question Text: Have you ever smoked tobacco in a hookah, even one or two puffs?]

- Yes
  *Branching Logic: when "Yes" selected, then:*
  **Do you smoke hookah…**[4]
  [Original Source Question Text: Do you now smoke hookah…]
    - Every day
    - Some days
    - Not at all
    - Don't know
    - Prefer not to answer

- No
- Don't Know
- Prefer Not To Answer

**Have you ever used smokeless tobacco products, even one or two times? (Smokeless tobacco products include snus pouches, Skoal Bandits, loose snus, moist snuff, dip, spit, and chewing tobacco.)**[4]
[Original Source Question Text: Have you ever used any of the following smokeless tobacco products, even one or two times? Choose all that apply.]

- Yes
  *Branching Logic: when "Yes" selected, then:*
  **Do you now use smokeless tobacco products…**[4]
  [Original Source Question Text: [Administrator Prompt] Has ever used smokeless tobacco, is/was a regular user, and uses every day or some days OR not at all.]
    - Every day
    - Some days
    - Not at all
    - Don't know
    - Prefer not to answer

- No
- Don't Know
- Prefer Not To Answer

---

**Thanks for your answers. The next questions will ask about drinking alcohol. This includes coolers, beer, wine, champagne, liquor such as whiskey, rum, gin, vodka, scotch, or liqueurs,**

**and also any other type of alcohol. This will help researchers better understand how alcohol affects health. As always, your answers are private.**

**In your entire life, have you had at least 1 drink of any kind of alcohol, not counting small tastes or sips? (By a "drink," we mean a can or bottle of beer, a glass of wine or a wine cooler, a shot of liquor, or a mixed drink with liquor in it.)**[5]
[Original Source Question Text: In your entire life, have you had at least 1 drink of any kind of alcohol, not counting small tastes or sips?]

- Yes
  *Branching Logic: when "Yes" selected, then:*
  **How often did you have a drink containing alcohol in the past year?**[6]
  [Original Source Question Text: How often do you have a drink containing alcohol?]
    - Never
    - Monthly or less
    - Two to four times a month
    - Two to three times a week
    - Four or more times a week
    - Prefer not to answer
      *Branching Logic: when anything other than "Never" or "Prefer not to answer'" selected, then:*
      **On a typical day when you drink, how many drinks do you have?**[6]
      [Original Source Question Text: How many standard drinks containing alcohol do you have on a typical day?]
        - 1 or 2
        - 3 or 4
        - 5 or 6
        - 7 to 9
        - 10 or more
        - Prefer not to answer

      **How often did you have six or more drinks on one occasion in the past year?**[6]
      [Original Source Question Text: How often do you have six or more drinks on one occasion?]
        - Less than monthly
        - Monthly
        - Weekly
        - Daily or almost daily
        - Never in the last year
        - Prefer not to answer
- No
- Prefer Not To Answer

**Thanks for your answers. Now we'd like to ask you about your experiences with medicines and other kinds of drugs. Some of the substances we'll talk about are prescribed by a doctor (like pain medications). We only want to know if you have taken them for reasons or in doses other than prescribed. We understand that these are sensitive questions. You may choose not to answer them. However, by providing answers, you are helping researchers better understand how these substances affect health.**

**In your LIFETIME, which of the following substances have you ever used?**[7]
[Original Source Question Text: In your LIFETIME, which of the following substances have you ever used?]

- ❑ Marijuana (cannabis, pot, grass, hash, weed, etc.)
  *Branching Logic: when "Marijuana (cannabis, pot, grass, hash, weed, etc.)" selected, then:*
  **In the PAST THREE MONTHS, how often have you used marijuana (cannabis, pot, grass, hash, etc.)?**[7]
  [Original Source Question Text: In the past three months, how often have you used the substances you mentioned (first drug, second drug, etc)?]
  - ○ Never
  - ○ Once or twice
  - ○ Monthly
  - ○ Weekly
  - ○ Daily or almost daily
  - ○ Prefer not to answer
- ❑ Cocaine (coke, crack, etc.)
  *Branching Logic: when "Cocaine (coke, crack, etc.)" selected, then:*
  **In the PAST THREE MONTHS, how often have you used cocaine (coke, crack, etc.)?**[7]
  [Original Source Question Text: In the past three months, how often have you used the substances you mentioned (first drug, second drug, etc)?]
  - ○ Never
  - ○ Once or twice
  - ○ Monthly
  - ○ Weekly
  - ○ Daily or almost daily
  - ○ Prefer not to answer
- ❑ Prescription stimulants for non-medical reasons (Ritalin, Concerta, Dexedrine, Adderall, diet pills, etc.)
  *Branching Logic: when "Prescription stimulants for non-medical reasons (Ritalin, Concerta, Dexedrine, Adderall, diet pills, etc.)" selected, then:*
  **In the PAST THREE MONTHS, how often have you used prescription stimulants for non-medical reasons (Vyvanse, Ritalin, Concerta, Dexedrine, Adderall, diet pills, etc.)**[7]

[Original Source Question Text: In the past three months, how often have you used the substances you mentioned (first drug, second drug, etc)?]

- ○ Never
- ○ Once or twice
- ○ Monthly
- ○ Weekly
- ○ Daily or almost daily
- ○ Prefer not to answer

❏ Other stimulants (methamphetamine, speed, crystal meth, ice, k2/spice, bath salts, etc.)

*Branching Logic: when "Other stimulants (methamphetamine, speed, crystal meth, ice, k2/spice, bath salts, etc.)" selected, then:*

**In the PAST THREE MONTHS, how often have you used other stimulants (methamphetamine, speed, crystal meth, ice, k2/spice, bath salts, etc.)**[7]

[Original Source Question Text: In the past three months, how often have you used the substances you mentioned (first drug, second drug, etc)?]

- ○ Never
- ○ Once or twice
- ○ Monthly
- ○ Weekly
- ○ Daily or almost daily
- ○ Prefer not to answer

❏ Inhalants (nitrous oxide, glue, gas, paint thinner, etc.)

*Branching Logic: when "Inhalants (nitrous oxide, glue, gas, paint thinner, etc.)" selected, then:*

**In the PAST THREE MONTHS, how often have you used inhalants (nitrous oxide, glue, gas, paint thinner, etc.)?**[7]

[Original Source Question Text: In the past three months, how often have you used the substances you mentioned (first drug, second drug, etc)?]

- ○ Never
- ○ Once or twice
- ○ Monthly
- ○ Weekly
- ○ Daily or almost daily
- ○ Prefer not to answer

❏ Sedatives or sleeping pills for non-medical reasons (Valium, Serepax, Ativan, Xanax, Librium, Rohypnol, GHB, etc.)

*Branching Logic: when "Sedatives or sleeping pills for non-medical reasons (Valium, Serepax, Ativan, Xanax, Librium, Rohypnol, GHB, etc.)" selected, then:*

**In the PAST THREE MONTHS, how often have you used sedatives or sleeping pills for non-medical reasons (Valium, Serepax, Ativan, Xanax, Librium, Rohypnol, GHB, etc.)?**[7]

[Original Source Question Text: In the past three months, how often have you used the substances you mentioned (first drug, second drug, etc)?]

- ○ Never
- ○ Once or twice
- ○ Monthly
- ○ Weekly
- ○ Daily or almost daily
- ○ Prefer not to answer

❑ Hallucinogens (LSD, acid, mushrooms, PCP, Special K, ecstasy, etc.)
*Branching Logic: when "Hallucinogens (LSD, acid, mushrooms, PCP, Special K, ecstasy, etc.)" selected, then:*
**In the PAST THREE MONTHS, how often have you used hallucinogens (LSD, acid, mushrooms, PCP, Special K, ecstasy, etc.)?**[7]
[Original Source Question Text: In the past three months, how often have you used the substances you mentioned (first drug, second drug, etc)?]

- ○ Never
- ○ Once or twice
- ○ Monthly
- ○ Weekly
- ○ Daily or almost daily
- ○ Prefer not to answer

❑ Street opioids (heroin, opium, etc.)
*Branching Logic: when "Street opioids (heroin, opium, etc.)" selected, then:*
**In the PAST THREE MONTHS, how often have you used street opioids (heroin, opium, etc.)?**[7]
[Original Source Question Text: In the past three months, how often have you used the substances you mentioned (first drug, second drug, etc)?]

- ○ Never
- ○ Once or twice
- ○ Monthly
- ○ Weekly
- ○ Daily or almost daily
- ○ Prefer not to answer

❑ Prescription opioids for non-medical reasons (fentanyl, oxycodone [OxyContin, Percocet], hydrocodone [Vicodin], methadone, buprenorphine, etc.)
*Branching Logic: when "Prescription opioids for non-medical reasons (fentanyl, oxycodone [OxyContin, Percocet], hydrocodone [Vicodin], methadone, buprenorphine, etc.)" selected, then:*
**In the PAST THREE MONTHS, how often have you used prescription opioids for non-medical reasons (obana, fentanyl, oxycodone [OxyContin, Percocet], hydrocodone [Vicodin], methadone, buprenorphine [Suboxone], etc.)**[7]

[Original Source Question Text: In the past three months, how often have you used the substances you mentioned (first drug, second drug, etc)?]

- ○ Never
- ○ Once or twice
- ○ Monthly
- ○ Weekly
- ○ Daily or almost daily
- ○ Prefer not to answer

- ❑ None of these drugs
- ❑ Prefer not to answer
- ❑ Other (Specify)

*Branching Logic: when "Other (Specify)" selected, then:*

**Please specify.**[8]

_______________

**In the PAST THREE MONTHS, how often have you used other drugs?**[7]

[Original Source Question Text: In the past three months, how often have you used the substances you mentioned (first drug, second drug, etc)?]

- ○ Never
- ○ Once or twice
- ○ Monthly
- ○ Weekly
- ○ Daily or almost daily
- ○ Prefer not to answer

**Thank you for answering these questions. We know they are very personal.**
**We want to remind you that your answers will only be shared with approved researchers.**
**Your privacy is very important to us.**

**Sources**

1. The Tobacco Use Supplement to the Current Population Survey. Bethesda, MD: National Cancer Institute: The Division of Cancer Control and Population Sciences (DCCPS). Available from: https://cancercontrol.cancer.gov/brp/tcrb/tus-cps
    a. Year of Original Source: 2003
    b. Brief Description of Source: The National Cancer Institute sponsors the Tobacco Use Supplement to the Current Population Survey (TUS-CPS). Since 1992, the U.S. Census Bureau has administered this survey every 3-4 years as part of the Current Population Survey. The collected data is used to monitor and evaluate tobacco control progress and conduct tobacco research.
2. Million Veteran Program (MVP). Washington DC: U.S. Department of Veteran Affairs. Available from: https://www.research.va.gov/mvp/
    a. Year of Original Source: 2011
    b. Brief Description of Source: The Million Veteran Program is an observational cohort study of the Department of Veterans Affairs that aims to advance research on how genes affect health and illness. One million veterans are being recruited to complete surveys, provide blood samples, and participate in a health assessment. Enrollment began in 2011 and is currently ongoing.
3. Prostate, Lung, Colorectal, and Ovarian Cancer Screening Trial (PLCO). Bethesda, MD: National Cancer Institute: Division of Cancer Prevention. 2014. Available from: https://prevention.cancer.gov/major-programs/prostate-lung-colorectal-and-ovarian-cancer-screening-trial
    a. Year of Original Source: 2008
    b. Brief Description of Source: The Prostate, Lung, Colorectal, and Ovarian (PLCO) Cancer Screening Trial is a large randomized trial established to assess the relationship between cancer-related mortality screening and numerous secondary endpoints. Data on men and women aged 55 to 74 was collected from 2006-2015. The trial was conducted by the National Cancer Institute.
4. PATH (Population Assessment of Tobacco and Health) Study. Bethesda, MD: National Institutes of Health. Available from: https://pathstudyinfo.nih.gov/landing
    a. Year of Original Source: 2015
    b. Brief Description of Source: The Population Assessment of Tobacco and Health (PATH) Study, started in 2013, is a national, longitudinal cohort study examining tobacco use and its effect on health. The PATH Study is a collaboration between the National Institute on Drug Abuse (NIDA), National Institutes of Health (NIH), and the Center for Tobacco Products (CTP) of the Food and Drug Administration (FDA).
5. National Epidemiologic Survey on Alcohol and Related Conditions-III (NESARC-III). Bethesda, MD: National Institute on Alcohol Abuse and Alcoholism (NIAAA). Available from: https://www.niaaa.nih.gov/research/nesarc-iii
    a. Year of Original Source: 2015
    b. Brief Description of Source: The National Epidemiologic Survey on Alcohol and Related Conditions (NESARC) is a cross-sectional survey conducted by the

National Institute on Alcohol Abuse and Alcoholism. The survey was started in 2011 and collects data on alcohol and drug use and associated risk factors and disabilities.

6. Instrument: AUDIT-C Questionnaire. Bethesda, MD: National Institute on Drug Abuse (NIDA). Available from: https://cde.drugabuse.gov/instrument/f229c68a-67ce-9a58-e040-bb89ad432be4
    a. Year of Original Source: 1998
    b. Brief Description of Source: The Alcohol Use Disorders Identification Test-C (AUDIT-C) is a three-item screening test for identifying hazardous drinkers and individuals with alcohol use disorders. It was developed in 1998 as a short form of the World Health Organization's ten-item AUDIT questionnaire.
7. NIDA-Modified Alcohol, Smoking, and Substance Involvement Screening Test. Bethesda, MD: National Institute on Drug Abuse (NIDA). Available from: https://datashare.nida.nih.gov/instrument/nida-modified-alcohol-smoking-and-substance-involvement-screening-test
    a. Year of Original Source: 2010
    b. Brief Description of Source: The National Institute on Drug Abuse-Modified Alcohol, Smoking, and Substance Involvement Screening Test (NM-ASSIST) is a screening tool of drug use in the primary and general medical care settings. NM-ASSIST was adapted in 2009 from the World Health Organization's Alcohol, Smoking and Substance Involvement Screening Test (ASSIST), Version 3.0.
