# Supplementary material for: Alcohol consumption among patients diagnosed with genitourinary cancers
Source: BJUI Compass. 2025 Sep 18;6(9):e70086. doi: 10.1002/bco2.70086 (PMC12446081; doi:10.1002/bco2.70086)
Supplement: Supplementary file 3 — Supplementary Table 2. Alcohol consumption patterns in the two‐month post‐diagnosis subcohort: Pan‐GU cancer versus noncancer controls. [file BCO2-6-e70086-s002.docx]

**Supplementary Table 2.** Alcohol consumption patterns in the two-month post-diagnosis subcohort: Pan-GU cancer versus noncancer controls.

|  | **Pan-GU Cancer Cohort (N=432)** | **Noncancer Cohort (N=1726)** | **OR (95% CI)^1^** |
| --- | --- | --- | --- |
| **Drinking Frequency** |  |  | 0.93 (0.66, 1.31) |
| *Never* | 102 ( 23.8) | 388 ( 22.6) |  |
| *Monthly or Less* | 113 ( 26.3) | 462 ( 27.0) |  |
| *2-4 per Month* | 77 ( 17.9) | 301 ( 17.6) |  |
| *2-3 per Week* | 62 ( 14.5) | 261 ( 15.2) |  |
| *4 or Greater per Week* | 75 ( 17.5) | 302 ( 17.6) |  |
| **Binge-Drinking Frequency** |  |  | 1.40 (0.96, 2.04) |
| *Never* | 199 ( 60.9) | 836 ( 63.8) |  |
| *<Monthly* | 82 ( 25.1) | 296 ( 22.6) |  |
| *Monthly* | 22 (  6.7) | 102 (  7.8) |  |
| *Weekly* | <20 | 55 (  4.2) |  |
| *Daily* | <20 | 22 (  1.7) |  |
| **AUDIT-C** | 2 (1,4) | 2 (1,4) | 0.95 (0.68, 1.33) |
| *1. Odds ratio (OR) and 95% confidence interval (CI) were calculated from ordinal logistic regression, with alcohol use variables treated as an ordinal dependent variable. Regression was adjusted for age, race, gender, smoking status, DMT2, hypertension, obesity, education status, and marital status.* | | | |
